# Cellular and transcriptional diversity over the course of human lactation

**DOI:** 10.1073/pnas.2121720119

**Published:** 2022-04-04

**Authors:** Sarah K. Nyquist, Patricia Gao, Tessa K. J. Haining, Michael R. Retchin, Yarden Golan, Riley S. Drake, Kellie Kolb, Benjamin E. Mead, Nadav Ahituv, Micaela E. Martinez, Alex K. Shalek, Bonnie Berger, Brittany A. Goods

**Affiliations:** ^a^Broad Institute of MIT and Harvard, Cambridge, MA 02142;; ^b^Program in Computational and Systems Biology, Massachusetts Institute of Technology; Cambridge, MA 02139;; ^c^Department of Chemistry and Institute for Medical Engineering & Science, Massachusetts Institute of Technology, Cambridge, MA 02139;; ^d^Computer Science and Artificial Intelligence Laboratory, Department of Mathematics, Massachusetts Institute of Technology, Cambridge, MA 02139;; ^e^Department of Bioengineering and Therapeutic Sciences, Institute for Human Genetics, University of California, San Francisco, CA 94143;; ^f^Ragon Institute of MGH, MIT, and Harvard, Cambridge, MA 02139;; ^g^Department of Biology, Emory University, Atlanta, GA 30322;; ^h^Koch Institute for Integrative Cancer Research, Massachusetts Institute of Technology, Cambridge, MA 02139;; ^i^Division of Health Science & Technology, Harvard Medical School, Boston, MA 02115;; ^j^Department of Immunology, Massachusetts General Hospital, Boston, MA 02114;; ^k^Thayer School of Engineering, Program in Quantitative Biomedical Sciences, Dartmouth College, Hanover, NH 03755

**Keywords:** single-cell RNA-sequencing, breast milk, mammary epithelial cell, macrophage, maternal health

## Abstract

Human breast milk (hBM) is a dynamic fluid that contains millions of cells, but their identities and phenotypic properties are poorly understood. We generated and analyzed single-cell RNA-sequencing (scRNA-seq) data to characterize the transcriptomes of cells from hBM across lactational time from 3 to 632 d postpartum in 15 donors. We found that the majority of cells in hBM are lactocytes, a specialized epithelial subset, and that cell-type frequencies shift over the course of lactation, yielding greater epithelial diversity at later points. Analysis of lactocytes reveals a continuum of cell states characterized by transcriptional changes in hormone-, growth factor-, and milk production-related pathways. Generalized additive models suggest that one subcluster, LC1 epithelial cells, increases as a function of time postpartum, daycare attendance, and the use of hormonal birth control. We identify several subclusters of macrophages in hBM that are enriched for tolerogenic functions, possibly playing a role in protecting the mammary gland during lactation. Our description of the cellular components of breast milk, their association with maternal–infant dyad metadata, and our quantification of alterations at the gene and pathway levels provide a detailed longitudinal picture of hBM cells across lactational time. This work paves the way for future investigations of how a potential division of cellular labor and differential hormone regulation might be leveraged therapeutically to support healthy lactation and potentially aid in milk production.

## Significance

Human breast milk is the nutritional food source evolved specifically to meet the needs of infants, but much remains to be learned about its composition and changes over the course of lactation. Our description of the cellular components of breast milk, their associations with maternal–infant dyad metadata, and quantification of alterations at the gene and pathway levels provide a longitudinal picture of human breast milk cells across lactational time. These results pave the way for improved therapeutic support of healthy lactation and milk production.

Human breast milk (hBM) is the nutritional food source evolved specifically to meet the needs of infants (1). Feeding exclusively with hBM is currently recommended for the first 6 mo of life, and this is one of the strongest preventative measures against mortality in children under 5 y old (2). In addition, breastfeeding has been linked to long-term health benefits for both infants and nursing mothers (1, 3, 4). Breastfed infants have decreased infections, improved gut and intestinal development, and improved regulation of weight long after termination of breastfeeding (5–7). Additionally, nursing mothers have a decreased risk of ovarian and breast cancers (8–10). Given that lactation and nursing provide substantial health benefits to mothers and infants, there is a need to better understand the molecular and cellular features of hBM, and broadly, how these may correlate with maternal and infant lifestyles and health. This understanding could lead to: insights into the functions of cells involved in lactation and how they may relate to decreased maternal milk production; actionable findings to inform physician advicse on the impact of lifestyle on breast milk composition; and, improved formulas and supplements to support changing infant health needs over the course of their first year of life.

The stages of lactation are canonically described as colostrum (0 to 3 d postpartum), transitional (6 to 14 d postpartum), and mature (>15 d postpartum) followed by involution, which begins within hours of the cessation of lactation (11, 12). During pregnancy, lactation, and involution, the human mammary gland undergoes drastic remodeling that requires coordinated shifts in tissue architecture and cellular composition guided by hormonal cues (13, 14). During lactation, the cells of the mammary gland are responsible for synthesizing and transporting the diverse components of hBM, as well as responding to maternal and infant signals to maintain lactational viability. A working knowledge of the cellular composition, activities, and regulation of the human mammary gland in the period between the establishment of lactation and involution is essential for understanding environmental factors that impact milk production, the responsiveness of the breast to the changing nutritional needs of the infant, and the mechanisms of long-term lactation. However, given the unique nature of this tissue niche, it is challenging to study lactating tissue directly in humans.

hBM contains live cells, which are thought to enter the breast through exfoliation during the process of breastfeeding, thereby providing an opportunity to study lactational cells (12). Cells from hBM are viable and can be cultured, and hBM immune cells were shown to transfer to offspring bloodstream and tissues in animal models (12, 15–17). The investigation of these live cells provides both noninvasive surveillance of the cells in the mammary mucosa and allows for a more detailed understanding of their roles in infant development (12, 17, 18). The cellular fraction of hBM contains both immune and somatic cells (11). Immune cell populations, such as macrophages, may be involved in the protection of the breast itself from infection during lactation (11, 18, 19). They may also produce important bioactive components, such as antibodies and cytokines, which play a role in the establishment of the infant immune system (20, 21). Somatic cells identified in breast milk include epithelial cells and a small fraction of stem cells (11). The dominant epithelial cell type in breast milk is secretory epithelial cells (lactocytes), which are involved in the synthesis of an array of factors, such as human milk oligosaccharides, lactose, micronutrients, fat, hormones, and cytokines, as well as their transport into the lumen of the lactating breast (11). Much remains to be learned about how the behaviors of lactocytes are regulated and the mechanisms by which they create these essential components and transport them into breast milk (11, 13, 22). Despite a dual role in conferring immunological protection and producing dynamic nutrition for infants, it is still unclear how the cellular composition of milk may change over the course of lactation (3, 4).

To date, several studies have used either bulk (12, 23–26) or single-cell RNA-sequencing (scRNA-seq) to study the transcriptome of hBM in small cohorts (15, 27). These studies have revealed subsets of epithelial cells in hBM, including progenitor luminal cells, and genes that change in bulk over the course of lactation. Bulk analysis, however, limits our ability to delineate key cell states and uncover specialized cell phenotypes (28, 29). Previous scRNA-seq analyses, meanwhile, havebeen limited by low sample numbers, small donor pools, and few time points, thereby decreasing the ability to characterize the cross-donor heterogeneity of breast milk longitudinally (15, 26, 30). Longitudinal studies of other milk factors have revealed dynamic shifts in hormone concentrations, cytokine levels, and overall protein content up to 3 mo postpartum, and suggest that most components decrease in concentration early in lactation (31–36). However, no transcriptomic studies to date have captured the full range of lactation across time.

**Fig. 1.**
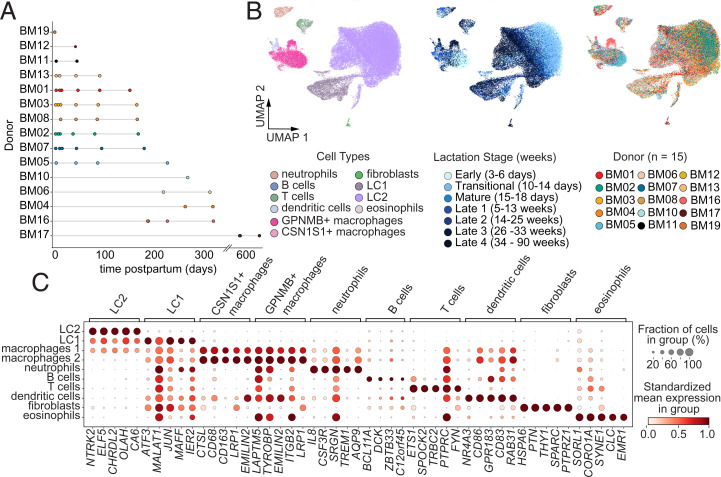
Atlas of cell types present in hBM across lactation. (*A*) Sampling timeline showing collection of samples for each donor as a function of time postpartum (days). (*B*) Projection of dimensionality reduced (UMAP) scRNA-seq data (*n* = 48,478 cells across 15 donors) colored by cell type, lactation stage (early, transitional, mature, and several late stages), and donor. (*C*) Marker genes (*x* axis and grouping labels on top) for each major cell type cluster (*y* axis). Circle size describes percent of cells in cluster expressing the gene. Color represents the mean log-normalized gene expression in that cluster standardized across clusters within each gene.

In order to better understand cellular dynamics and longitudinal lactational heterogeneity, we sought to characterize the transcriptomics of hBM-derived cells using scRNA-seq on longitudinal samples. hBM from 15 human donors was profiled longitudinally across various stages of lactation (Fig. 1*A* and *SI Appendix*, Table S1). For each sample, we also collected a rich set of information about the mother–infant dyad, including vaccine history, illness, and daycare status. Our results provide a valuable single-cell characterization of hBM-resident cells over the course of lactation, with a dataset comprised of over 48,478 cells from 50 samples (Dataset S1). Computational analysis of this data identified key cell subsets, including immune cells and epithelial cells at each lactation stage. Through additional analyses, we find that health and lifestyle changes, including the use of hormonal birth control and the start of daycare, may be associated with alterations in cell frequencies over lactation. We also nominate pathways and genes that are altered in epithelial subsets over the course of lactation, including those that may be hormonally regulated. Taken together, our data and analyses provide a longitudinal characterization of single cells in breast milk and shed light on the gene programs that may drive crucial human lactocyte functions over the course of lactation.

## Results

### We Identify Major Cell Types of the Breast Epithelium and Immune Cells in hBM over the Course of Lactation.

We first optimized a process for generating scRNA-seq data from cells in hBM. Previous studies characterized how sample handling, as well as methods used for cell isolation, can significantly impact the transcriptomes of isolated cells (37, 38). We compared several workflows for upstream handing of collected hBM—including fresh isolation of cells, holding at 4 °C overnight until cell isolation, and a single freeze/thaw of whole milk before isolating cells—as well as several methods for isolating cells, including sorting live cells, live cell enrichment with a bead-based kit, or centrifugal isolation of fresh cells as previously described (*SI Appendix*, Fig. S1) (39). We found that for each method, except for freezing, quality-control metrics were comparable, and we identified expected cell types in milk, including epithelial and immune cell subsets (*SI Appendix*, Fig. S1 *B* and *C*). Fresh processed cells, sorted cells, or live-enriched cells clustered together in principal component (PC) space, suggesting little gain by additional processing prior to performing scRNA-seq. Additionally, we found that in one donor, fresh but not frozen processing allowed us to retain macrophages (*SI Appendix*, Fig. S1*D*). In agreement with previous studies, we found that isolation of cells from fresh milk resulted in the highest-quality data and we therefore used this method for our samples analysis (40).

To better understand the transcriptomes of single cells in hBM over the course of lactation, we recruited donors to provide milk samples at several time points postpartum, including colostrum/early (3 to 6 d), transitional (10 to 14 d), mature (15 to 18 d), and several late points postpartum (5 to 90 wk) (Fig. 1*A*). We performed Seq-Well S^3^ with freshly isolated cells from whole milk to generate high-quality single-cell transcriptomic data across all lactation stages (*SI Appendix*, Fig. S2*A*).

**Fig. 2.**
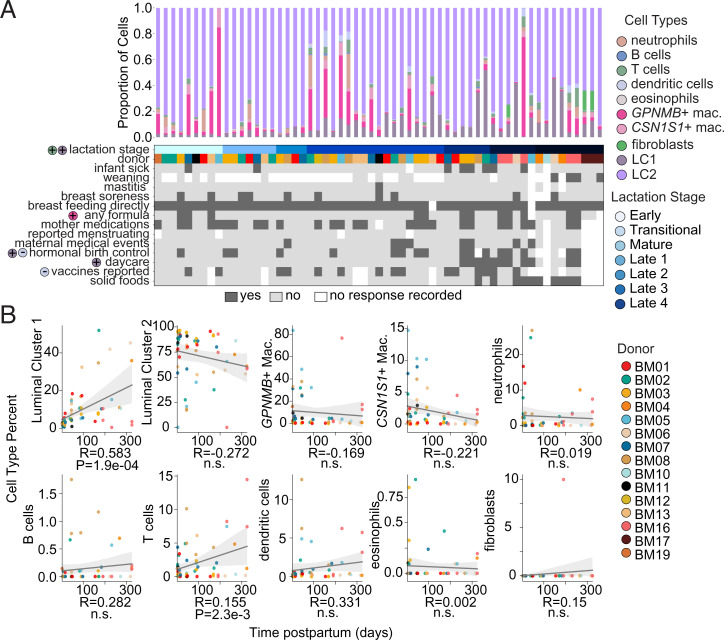
Frequency of cell types over the course of lactation. (*A*) Frequency of cell types identified for each sample (*Upper*) and associated maternal and infant health information metadata (*Lower*) collected in user-reported questionnaires. Colored circles to the left of metadata names indicate associations of metadata with cell-type (specified by color) abundances and the direction of the association via plus (+) or minus (−). Different donors show associations in different directions with cell-type’s proportions (*SI Appendix*, Table S2) (*B*) Normalized cell frequencies as function of time postpartum for samples at timepoints <400 d postpartum are shown for all identified cell types. Spearman correlation coefficients (*R*) and *P* values from generalized additive models are shown below each plot, and confidence intervals around Spearman correlations are displayed in gray.

We performed unsupervised clustering across all high-quality cells and labeled cell types using marker genes (Fig. 1 *B* and *C* and Dataset S2) previously identified in the context of the mammary gland and the immune system (41–43). Our analyses revealed 10 broad cell types representing both epithelial and immune cell compartments (Fig. 1*B* and *SI Appendix*, Fig. S2*B*). We identified seven top-level immune cell clusters, including B cells (*TCF4*, *SEL1L3*, *CCDC50*), dendritic cells (*NR4A3*, *REL*), T cells (*ETS1*), two macrophage clusters (*GPNMB*^+^ macrophages [*CD68*, *GPNMB*, *CTSL*], and *CSN1S1*^+^ macrophages [*CD68*, *CSN1S1*, *XDH*]), neutrophils (*IL8*, *CSF3R*), and eosinophils (*SORL1*, *CORO1A*). We also identified three nonimmune top-level clusters, including luminal cluster 2 (LC2) (*XDH*, *CSN1S1*, *CSN3*), luminal cluster 1 (LC1) (*CLDN4*, *JUN*, *KLF6*), and fibroblasts (*SERPINH1*, *PTN*). Interestingly, we found that these cells clustered predominantly by cell type, rather than donor, suggesting that donor-to-donor differences were not the primary axis of variation. These subsets agree with other datasets describing scRNA-seq on hBM in smaller cohorts on the basis of component cell clusters and genes expressed in these clusters with some exceptions (*SI Appendix*, Fig. S3 *A*–*E*) (15, 27, 30). Due to increased cell numbers in this dataset, we identified more immune clusters (seven) than previous studies, which identified two or three clusters, adding dendritic cells, a second macrophage cluster, and eosinophils to these previously identified. We named our two large epithelial clusters as LC in accordance with previous studies since both express high levels of milk synthesis genes (i.e., *LALBA* and *CSNs*); however, LC2 expresses these genes at higher levels, and LC1 expresses higher levels of distinct genes (Fig. 1*C* and *SI Appendix*, Fig. S3 *C* and *D*) (30). Unlike previous studies, we identified a small cluster of fibroblast-like cells in hBM expressing genes, consistent with fibroblasts found in breast tissue datasets (*COL1A1*, *DCN*, *FN1*) (*SI Appendix*, Fig. S3 *A* and *F*). This small cluster contains cells from only two donors at very late milk stages, so further study is required to understand if these cells are found in hBM at various lactational stages (Fig. 2*A*). Overall, lactocyte epithelial cells (LC1 and LC2) were the most abundant cell type across both donor and lactation stage (mean 81.7% of all cells per sample, SD 24%), with macrophages comprising the most abundant immune cell type (50.5% of immune cells per sample, SD 34%) (Fig. 2*A*).

### Cell Frequencies Are Dynamic over the Course of Lactation and Associate with Maternal–Infant Metadata.

In order to better understand longitudinal variation in hBM-derived cells and our cohort metadata, we plotted total cell counts, cell-type frequencies, and metadata for each sample in our cohort (Fig. 2*A*). We found that the total cell counts per milliliter of milk decreased over the course of lactation, agreeing with previous literature showing a decrease in total cell counts in mature milk (*SI Appendix*, Fig. S4) (23). We also found that the majority of our cohort were directly breastfeeding, with five donors (nine samples) additionally supplementing with formula and six donors (nine samples) reporting supplementation with solid foods. Several donors reported breast soreness periodically over the course of the study, with only one donor reporting mastitis at sample collection (Dataset S1). Additionally, none of our donors reported menstruating at the time of sample donation and four were on hormonal birth controls or other reported medications. Finally, we had three donors who had begun weaning and six whose children had started daycare during our study. Globally, the variability in reported metadata allowed us to interrogate how cellular composition may be impacted by shifts in time, lifestyle, and maternal or infant health status. We noted that a substantial amount of variability in these cell compositions may be attributed to individual donors, with a single donor consistently showing substantially larger macrophage proportions (BM05) and all of the fibroblast cells coming from two donors (BM16, BM17) (Fig. 2*A*).

We tested the association between the abundance of identified cell types with any reported metadata using generalized additive models (*SI Appendix*, Table S2). While we found that nothing was significantly associated following correction for multiple hypotheses, we did find some associations indicating potential heterogeneity. We found *GPNMB*^+^ macrophage proportion associated with formula supplementation, LC1 proportion positively associated with daycare attendance and with use of hormonal birth control, and dendritic cell proportion negatively associated with use of hormonal birth control and with infant vaccinations (Fig. 2*A* and *SI Appendix*, Table S2). Since it is unclear if we would expect the proportions of epithelial and immune cells to respond to each of these covariates in coordinated ways, we also tested for associations between donor metadata and immune subsets as a proportion of only immune cells (*SI Appendix*, Table S2). While these associations still did not meet our multiple testing correction threshold, we found that eosinophil proportion was negatively associated with formula use, dendritic cell proportion, positively associated with infant illness, and that *GPNMB*^+^ macrophage proportion was negatively associated with infant or maternal vaccination reports. We acknowledge that given our study design, often donor is conflated with certain metadata features as well as time, but include these tentative associations to motivate further studies in larger cohorts designed prospectively.

We next sought to refine our understanding of which cell types were correlated with time postpartum by looking at associations of cellular proportions with time postpartum. We found that several cell types remained relatively consistent over the sampled course of lactation, including LC2 and macrophages (Fig. 2*B*). We also found several cell types that were significantly positively associated with time postpartum, including LC1 (*P* = 1.9e-4) and T cells (*P* = 2.3e-3) (Fig. 2*B*). As with our metadata, we tested for associations between time postpartum and immune cells represented as a proportion of only immune cells and of epithelial cells as a proportion of just epithelial cells (*SI Appendix*, Fig. S5 *A*–*D* and Table S2). With this representation, LC1 proportion was still significantly associated (*P* = 3.5e-3) but T cells were not. Alterations in the composition of the epithelial compartment may suggest some emergent cellular functions that support later lactation, and the presence of more T cells, while still very low fractions of total immune cells, could reflect increasing infant or maternal illnesses reported at later time points in our cohort.

### Macrophages in hBM Have Unique Transcriptional and Functional Programs.

**Fig. 3.**
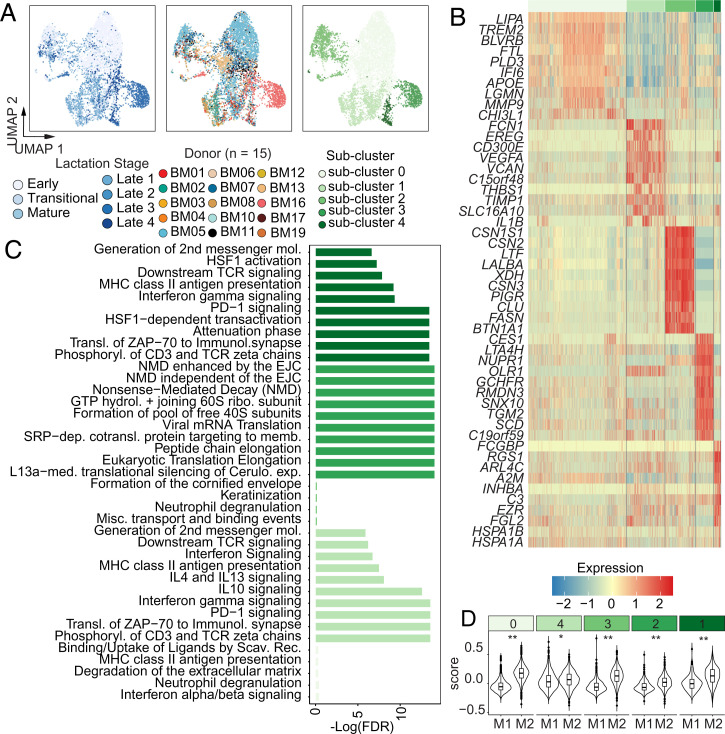
Macrophage subclusters across lactation stage. (*A*) UMAP projection of hBM-macrophages, colored by lactation stage (*Left*), donor (*Center*), and macrophage subcluster (*Right*). (*B*) Heatmap of top marker genes for each identified macrophage subcluster (Dataset S3). (*C*) Reactome enrichment results for each subcluster. Full results are shown in Dataset S4. (*D*) Module scoring results for M1 or M2 gene sets for each subcluster (**P < 2.2e-16, *P < 2.3e-6).

We found that the majority of immune cells in hBM over the course of lactation were macrophages, agreeing with previous literature (44). We next sought to better understand the potential functions and phenotypes of macrophages in hBM given that their percentages were altered in response to formula supplementation. We performed subclustering analyses and functional enrichment of marker genes that were identified for each subcluster (Fig. 3 *A* and *B*, *SI Appendix*, Fig. S6, and Datasets S3–S5). We found five subclusters of macrophages that spanned lactation stage, with macrophage subcluster 0 predominantly identified at early milk stages and macrophage subcluster 3 predominantly arising from donor BM16 (*SI Appendix*, Fig. S5*E*). Macrophage subclusters were defined by distinct gene signatures and pathway enrichment results (Fig. 3 *B* and *C*). Macrophage subclusters 0, 1, and 4 were defined by pathways related to interactions with T cells, neutrophils, and immune tolerance, including interleukin (IL)-10 and PD-1 related pathways. These enrichments were driven by unique sets of genes present in each subcluster (Dataset S4). Interestingly, macrophage subcluster 0 was defined by several marker genes characteristic of lipid-associated macrophages (*LIPA*, *TREM2*) and those involved in iron regulation (*FTL*) (45). Macrophage subcluster 3 was enriched for several translation-related pathways, and defined by lipid-related genes like *SCD* and *LTA4H*, and stress-response genes like *NUPR1*. We caution that this subcluster was predominantly comprised of one donor, BM16, and thus may reflect specific variations in myeloid cell state related to that particular donor. Finally, macrophage subcluster 2, which was comprised almost entirely of *CSN1S1*^+^ macrophages, was defined by structural pathways, transport, and keratinization. This may suggest that these macrophages are important for structural maintenance or have altered their transcriptional state in response to their local tissue milieu, possibly via phagocytosis (46). Future work should explore these mechanisms since hBM components have been shown to promote tolerogenic phenotypes in myeloid cells (47, 48).

In order to determine if macrophages in each cluster were more inflammatory (M1) or antiinflammatory (M2) in nature, we scored these clusters for M1 or M2 gene signatures (45, 49). While it is widely recognized that macrophages adopt a diverse array of phenotypes in the context of tissues, conventional M1 or M2 status is a useful indicator and comparison point to existing literature in the context of the lactating mammary gland (18, 42). To accomplish this, we generated module scores for M1 or M2 gene sets within each macrophage subcluster. Overall, each subcluster, except for subcluster 1, scored higher for M2-gene sets, suggesting that the majority of macrophages in hBM are M2-like (Fig. 3*D*). Combined with our enrichment results, and previous literature reports in the context of the mammary gland, this suggests that macrophages in hBM predominantly serve immunosuppressive and tissue maintenance functions (18, 50).

Finally, we determined if sample distribution across clusters varied with four metadata variables of interest, including infant medical events, weaning status, daycare status, and supplementation with formula (*SI Appendix*, Fig. S7). We found that subcluster 0 had the highest proportion of reported infant medical events, which included both vaccines and illness. Second, we found that weaning-derived macrophages were predominantly found in subclusters 4, 0, and 2 (*SI Appendix*, Fig. S7). Future work should address the functional changes in macrophages in hBM postweaning, since it is known that macrophages shift their transcriptional and functional phenotypes dramatically in response to alterations in the mammary gland (18, 50).

### Epithelial Cell Subclusters in hBM Are Enriched for Distinct Functions and Diversify over the Course of Lactation.

**Fig. 4.**
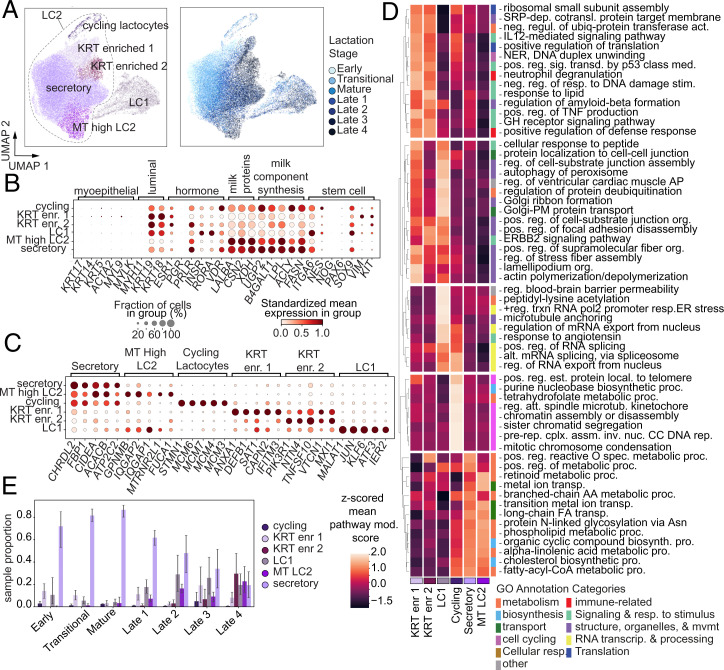
Subclustering analysis of epithelial cells reveals an increase in epithelial diversity over the course of lactation. (*A*) UMAP visualization of epithelial cells colored by epithelial subcluster (*Left*) or donor (*Right*). (*B*) Mean expression in cell subset standardized within genes (color) and percent of cells expression (dot size) of canonical mammary epithelial marker genes in each epithelial subgroup. (*C*) Mean expression in cell subset standardized within genes (color) and percent of cells expression (dot size) of marker genes for each epithelial subgroup identified by pseudobulk marker gene identification. (*D*) Reduced top Enrichr results from the GO biological processes 2021 database on the marker genes for each subgroup, colored by the mean gene-set score for all genes in that pathway on cells in that subgroup, scaled by a *z*-score across subgroups. GO annotation categories are labeled by manual curation of related GO classifications. (*E*) Proportions of each subgroup per sample, split by milk stage. Error bars show SD.

In order to better understand the full heterogeneity of epithelial cells in hBM over the course of lactation, we performed subclustering analysis on the epithelial cells (*Materials and Methods* and *SI Appendix*, *Supplementary Methods*). We identified six subclusters of epithelial cells (Fig. 4*A*, *SI Appendix*, Figs. S8 and S9, and Dataset S6). The breast epithelium is composed of epithelial cells derived from several lineages, including basal/myoepithelial cells and luminal cells. Consistent with other scRNA-seq studies of hBM, we did not identify cells expressing basal/myoepithelial lineage markers identified by scRNA-Seq of nonlactating human breast tissue (Fig. 4*B* and *SI Appendix*, Figs. S3 *B*, *C*, and *F* and S8*F*) (15, 27). We found that all epithelial subclusters expressed genes related to milk synthesis—such as *LALBA*, *CSN2*, *XDH*, and *FASN*, as well as canonical luminal cell markers (*EPCAM*, *KRT18*, *KRT19*)—suggesting a clear luminal lineage and role in milk production (Fig. 4*B*, *SI Appendix*, Fig. S3 *B*, *C*, and *F*, and Dataset S6) (12, 27). Previous studies on the nonlactating breast have distinguished several luminal cell signatures, including luminal progenitors (LP), secretory luminal, and hormone responsive (HR^+^) luminal cells. Consistent with previous scRNA-seq studies of hBM, we did not identify a discrete stem cell or HR^+^ cluster, but found that both major epithelial cell clusters, LC1 and LC2, expressed some progenitor markers (*SOX9*, *ITGA6*) and hormone receptor markers (*PRLR*, *INSR*, and *ESR1*) in subsets of their cells (Fig. 4*B* and *SI Appendix*, Figs. S2 and S3 *B*, *C,* and *F*).

In order to better understand the functions of each subcluster, we identified marker genes (Fig. 4*C*) and performed enrichment analyses (Fig. 4*D*). The largest subcluster of epithelial cells, secretory lactocytes, expressed the highest levels of secretory markers (*CHRDL2*, *CIDEA*, *ATP2C2*), and lipid and lactose synthesis genes (*FBP1*, *ACACB*) (Fig. 4*C*). This cluster was also enriched for pathways associated with metabolic processes, transport, and biosynthesis (Fig. 4*D*). While there is significant heterogeneity within this large group of cells, gene expression variability changed continuously with time and not according to discrete subcluster groups (*Materials and Methods*, Fig. 4*A*, and *SI Appendix*, Fig. S8*A*). The second largest subcluster, LC1 cells, was defined by expression of AP-1 transcription factor subunits (*JUN*, *ATF3*, *FOS*), as well as *MALAT1*, *KLF6*, and *CLDN4*, genes involved in tight junction pathways (51). This subcluster was enriched for pathways related to microtubule and cellular organization (microtubule anchoring, actin polymerization or depolymerization), cell–cell junction assembly, protein transport via the Golgi, and ERBB2 signaling pointing to an involvement in the establishment and maintenance of the cell–cell tight junctions, which structurally support the alveolar structures in the lactating breast (Fig. 4*D*) (52).

The cycling epithelial subcluster was defined by the expression of cell-cycle genes (*STMN1*, *TOP2A*) and was enriched for cell-cycle–related processes, as well as several metabolic processes. This subcluster is also composed entirely of cells whose cell-cycle score indicated they were in the G2M and S phases (*SI Appendix*, Fig. S8*D*). The MT-high LC2 cluster was defined by similar gene expression to the secretory epithelial cells but with higher mitochondrial gene proportion than other subclusters of the LC2 cluster (*SI Appendix*, Fig. S8*I*). While mitochondrial RNA percentage is often used as a metric for dead or dying cells in scRNA-seq analysis, we maintained this cluster in the dataset because it met our very conservative threshold for mitochondrial RNA percentage, showed an interesting trend of increasing proportion over time, and may relate to altered metabolic activity in these cells (53).

The KRT high lactocyte 1 cluster was defined by expression of cytoskeleton and structural genes (*S100A9*, *KRT15*, *KRT8*, *VIM*) as well as immune response genes (*ANXA1*, *DEB1*, *IFITM3*, *CD74*, *HLA-B*). This subcluster is enriched in pathways broadly related to translation, positive regulation of defense response, and several signaling pathways (Fig. 4*D*). The KRT high lactocyte 2 subcluster was enriched for similar pathways to the KRT high lactocyte 1 group, but this subcluster shared fewer high-scoring pathways with the LC1 subcluster, suggesting more of a supporting role in milk production and less of a structural role.

Finally, we determined how these subclusters were changing in proportion as a function of lactation stage (Fig. 4*E* and *SI Appendix*, Fig. S5 *A* and *C*). Globally, we found that the cellular composition of later lactational timepoints was more diverse, with higher Shannon entropy values per sample, as compared to earlier time points, where early time points are dominated by secretory epithelial cells (*SI Appendix*, Fig. S8*K*). All subclusters, except the secretory and the cycling lactocytes, increased over the course of lactation. This may indicate that some degree of cellular specification is acquired over the course of lactation, potentially to meet changing demands on the maternal–infant dyad. For example, the increase in mitochondrial activity in the MT high LC2 subcluster, coupled with alterations in several metabolic pathways, may suggest that there are altered metabolic programs that support the high lactational demand and tissue turnover in later lactation.

### There Were Significant Changes in Gene Expression over the Course of Lactation in the LC1 Epithelial and Secretory Lactocyte Subclusters.

**Fig. 5.**
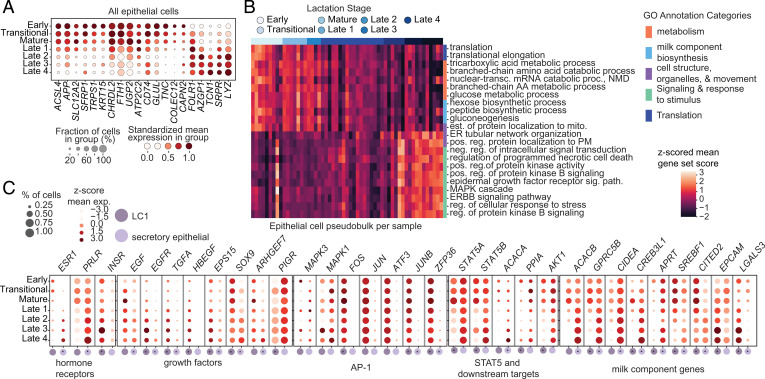
Transcriptional programs of luminal epithelial cells change over the course of lactation. (*A*) Genes of interest changing over all epithelial clusters over the course of lactation, standardized expression over time; full results in Dataset S8. (*B*) Reduced top Enrichr GO biological process results on genes changing over time shared across both LC1 epithelial cells and secretory epithelial cells. Heatmaps represent sample means of gene-set scores of each pathway (rows) *z*-scored across samples (columns) and samples ordered by increasing time postpartum. Pathways colored by curated related GO term classifications; full results are in Dataset S9. (*C*) Hormone receptors, growth factor pathway components, AP-1 subunits, STAT5 and downstream targets, and milk component genes change with different dynamics in the LC1 epithelial and secretory lactocyte subclusters. Plots colored by mean expression of cells in each milk stage and time point *z*-scored across all time points and both subgroups (an asterisk indicates adjusted *P* < 0.05 via DESeq2 analysis over time) (*Materials and Methods*). Full results are in Dataset S8.

We found that both the fractional abundance of epilthelial cells and their overall diversity increased with time postpartum in hBM, so we next asked which genes and pathways also changed over the course of lactation in epithelial cells. To accomplish this, we performed differential expression with pseudobulk populations across time postpartum within each epithelial subcluster and annotated genes and pathways, which changed across all epithelial subclusters as well as those that changed in a single subcluster and several that changed in opposite directions in different subclusters (*Materials and Methods* and Datasets S7, S8, and S9). We found that there were many genes that were differentially expressed over time across all epithelial cells, including several that decreased over time—such as *APP*, *KRT15*, and *FTH1*—and several that increased, such as *LYZ* and *TCN1* (Fig. 5*A*). Lysozyme, encoded by the transcript *LYZ*, one of the most abundant bioactive components of milk, has previously been shown to increase in later stages of lactation (54). Broadly, genes in pathways related to metabolism and milk component biosynthesis decreased in expression over the course of lactation while genes in structural pathways and those involved in signaling and response to stimulus increased in expression over the course of lactation (Fig. 5*B*).

The LC1 and secretory lactocyte subclusters had the largest numbers of genes differentially expressed over lactation time unique to the subclusters (Dataset S7). Enrichment analyses of these differentially expressed genes (*Materials and Methods*) identified both shared and distinct pathways that changed with time in both cell subclusters (Fig. 5*B* and *SI Appendix*, Fig. S10). In secretory lactocytes, genes with decreasing expression over time were enriched for pathways related to milk component metabolism and milk component biosynthesis, translation, and cellular respiration, while the genes that increased in expression over time were enriched for pathways involved in milk component transport, as well as transcription (*SI Appendix*, Fig. S10*A*). The cholesterol biosynthesis pathway was enriched in time-varying genes from both cell subclusters, but its expression score increased over time in secretory lactocytes and decreased over the course of lactation in the LC1 subcluster (*SI Appendix*, Fig. S10*B*). Additionally, over the course of lactation, pathway scores for TGF-β signaling, chromatin remodeling factors, cytoskeletal transport, vesicle-mediated transport, and apoptosis all increased in LC1 cells with time postpartum (*SI Appendix*, Fig. S10*B*). Taking these data together, we identified many pathways that are differentially altered with lactation time in the major subclusters of epithelial cells.

In order to nominate key genes and factors that might be responsible for pathway-level changes in these two subclusters, we looked at the expression of key regulators that were differentially expressed with time postpartum, including those important for hormone signaling, growth factor signaling, AP-1 signaling, factors involved in STAT5 signaling, and several milk production component genes (Fig. 5*C*) (55–57). We found that the expression of several hormone receptor genes changed in opposite directions with time in the LC1 and secretory lactocyte subclusters, with estrogen receptor (*ESR1*) and prolactin receptor (*PRLR*) increased in secretory lactocytes and decreased in LC1 cells. Insulin receptor (*INSR*) increased in just LC1 cells. These opposite changes in the *PRLR* receptor are accompanied by corresponding changes in prolactin-regulated *STAT5*, a core lactational gene involved in proliferation, cell survival, and milk component synthesis, and related pathways over the course of lactation (55, 58–63). In LC1 cells, we observed decreases in *STAT5A/B* expression and downstream targets, such as *AKT1*, *ACACA* (a gene involved in fatty acid synthesis), and *CSN2* (the gene encoding β-casein) over the course of lactation (Fig. 5*C*) (57). We also found a decrease in the gene ontology (GO) terms cellular macromolecule biosynthetic process and cholesterol biosynthetic process in LC1 cells over the course of lactation, all of which are related to milk component synthesis and could be prolactin-regulated via *STAT5* (*SI Appendix*, Fig. S10*B*) (58, 61, 63, 64). Expression of *PRLR* changed in the opposite direction in secretory epithelial cells, increasing with time postpartum along with some increase in *JAK2*, *STAT5A*, and target *ACACA* expression (Fig. 5*C*). Given that these receptors are crucial to orchestrating the functions and tissue structure of the lactating mammary gland, our data suggest that these two subclusters may differentially contribute to these functions over time in a hormonally regulated manner.

## Discussion

In this study, we used scRNA-seq to provide an in-depth characterization of transcriptional changes over the course of lactation in hBM at single-cell resolution. Our cohort represented a range of experiences of maternal–infant dyads allowing us to determine how cellular content varied over the course of lactation, examine which maternal and infant factors (metadata features) correlated with hBM cellular content, how cells changed their transcriptomes longitudinally, and what the full depth of cellular diversity was over each lactation stage. To our knowledge, our study is unique in correlating maternal–infant dyad metadata with cell proportions over the full course of lactation. The relatively small size of our donor cohort compared to the number of metadata variables considered limits the strength of these associations. We expect that further study on additional cohorts combined with these observations will lead to important advances in the understanding of the impact of the behavior and health of the maternal–infant dyad on breast milk cellular composition.

We found that the majority of immune cells in our data were macrophages and that adaptive immune cells, including T cells and B cells, were only a small fraction of the total recovered cells from hBM. Our data suggest that milk is dynamic over the full course of lactation, with different immune cells expanding and contracting within each sample over time. Previous reports have defined infiltration of CD45^+^ cells in response to mastitis and other infections, and have characterized extensively the features of immune cells by canonical makers in the context of preterm birth or infection (3, 17, 23, 44, 65). These studies predominantly relied on flow cytometry, and here, we were able to use scRNA-seq to characterize alterations in cellular composition in depth with less potential bias. Our limited sample processing (e.g., no staining or sorting) may have enabled us to recover more macrophages than previous studies. Our top-level clustering revealed two major populations of macrophages, both enriched for canonical macrophage markers like *CD68*. We found that our *CSN1S1*^+^ macrophage cluster was enriched for several milk production transcripts, like *CSN*. These could be present in this population as “passenger” transcripts that originate from engulfed apoptotic bodies, or these may be functionally important given that previously defined ductal associated macrophages expressed similar milk-related transcripts (18, 66). We also identified several subclusters of macrophages, and our GO enrichment and module scoring analyses suggest that these may be more tolerogenic in nature. Previous reports in mice have observed extensive diversity in mammary duct macrophages, and have found that these cells alter their transcriptomes significantly over reproductive cycles (18). This, coupled with work in the context of breast cancer and pan tissue analyses, suggests that the full functional diversity of macrophages in the human breast has yet to be fully characterized (67). Future work should seek to better understand the factors, whether tissue- or milk-specific, that promote tolerogenic functions of macrophages during lactation, and what secreted factors from macrophages might support healthy mammary gland functions. The putative association of our macrophage *GPNMB*^+^ cluster with formula supplementation motivates questions about how formula supplementation might alter cellular composition in hBM, and whether this could impact the functions of hBM-derived macrophages.

We identified two major populations of epithelial cells (LC1 and LC2) as well as several subclusters of LC2 cells (cycling lactocytes, KRT enriched 1, KRT enriched 2, secretory, MT high LC2). While the major populations agree with previous reports, our data demonstrate the functional diversity of these cells and differences compared to their breast tissue counterparts (15). Our data suggest that LC1 cells may provide more structural support during later lactation stages, while LC2 cells and its associated subclusters may produce more milk components. Consistent with previous work, we also did not see cells expressing genes expected from myoepithelial, basal, or stem cells (15, 27).

In addition to being associated with time postpartum, the proportional abundance of LC1 cells were positively associated with two external factors: daycare attendance and hormonal birth control usage. The effect of these variables is challenging to disentangle in our dataset due to their correlation with time postpartum, but our results suggest that future work should seek to understand how external perturbations and behaviors, potentially including increased pumping frequency and circulating hormone levels, impact the mammary gland specifically during later stages of lactation. Our differential expression results identifying key growth factors and hormone receptors, like *ESR1* and *INSR*, that changed in expression over time in these cells suggest that these may be hormonally regulated and emerge as important structural cells in later stages of lactation.

At the gene level, bulk transcriptomic studies have shown transcriptional changes between colostrum, transitional, and mature milk in pathways presumed to originate from epithelial cells, indicating that insulin signaling, lactose synthesis, and fatty acid synthesis pathways increase during these early stages of lactation. Only a few transcriptional studies have characterized the gene-expression changes during later stages of lactation before involution, suggesting higher expression levels of *PRLR*, *STAT5A*, and milk protein and lipid synthesis genes during lactation; however, bulk studies have not had the resolution to describe changes in which cells expressi these genes (12, 25). We identify the epithelial cell subclusters in which key genes are changing across both time and many donors, allowing us to gain insights into potential alterations in milk transport, synthesis, and production. Previous studies suggested that more milk components are transferred from the blood to the milk via tight junctions at later time points in lactation and fewer components are synthesized in the lactocytes themselves (68). Our results suggest that this functional change may come from changes in the proportions of cells executing these functions over the course of lactation. The LC1 cell cluster, whose marker genes are enriched for genes involved in tight junctions, increased in abundance over the course of lactation, while we saw a decrease in the proportional abundance of the secretory lactocyte subcluster whose core enriched functions involve milk component synthesis and secretion. We also saw a decrease in milk component synthesis-related genes (*UGP2*, *CHRDL2*) (Fig. 5*A*) and a decrease in the GO terms gluconeogenesis, hexose biosynthetic process, and glucose metabolic process over time in both clusters (Fig. 5*B*). This might suggest a decrease in transcription of milk component-related genes over the course of lactation and agrees with previous studies that have shown a linear decrease in overall protein concentration and decreased human milk oligosaccharides and lactose synthesis over the course of lactation (68–70).

Due to our long follow-up study, we were able to capture late stages of mature milk (late 2 to late 4), possibly observing alterations in epithelial cells to meet altered demands as babies begin to eat solid foods. Increased cellular specialization and altered abundance of epithelial subclusters that we describe may provide mechanistic insights into changes in the maintenance of milk secretion over the course of lactation. Future work should seek to understand how this relates to milk component production and synthesis in the mammary gland, transport from maternal serum, or milk volume production. Validation of these findings in breast tissue is also required to disentangle the potential impact of changes in epithelial composition of hBM over the course of lactation due to changes in exfoliation dynamics during pumping, possibly in addition to actual changes in breast tissue composition.

We also found that pathways downstream of several hormone receptors—including prolactin signaling, estrogen signaling, and human growth factor signaling—were enriched in the marker genes of the LC2 cells, indicating that these cells are likely directly hormonally regulated. Hormones in hBM serve both as regulators of the mammary gland itself, as well as bioactive components passed to the infant. The LC1 and secretory epithelial cell subclusters showed opposite changes in expression of several hormone receptors known to be important for regulating lactogenesis and involution, as well as their downstream targets over the course of lactation (Fig. 5*C*). This may suggest a possible regulatory mechanism of these synthesis and transport changes *vis a vis* a division of labor between cell types over the course of lactation. The increase in *PRLR* expression and prolactin-regulated targets in secretory epithelial cells and opposite decrease in LC1 subclusters could explain other differential functions of these cell subclusters over the course of lactation if, for example, the LC1 cells become more specialized over the course of lactation and less responsible for milk component synthesis over the course of lactation, with decreased responsiveness to prolactin and decreased JAK2/STAT5-regulated milk component synthesis. We see similar alterations in the dynamic expression of several growth factors that regulate milk production and secretion, like *EGF* (71). Further studies should investigate this division of cellular labor and consider the direction of this regulation and how it might be leveraged therapeutically to potentially aid in milk production.

Our description of the cellular components of breast milk over the course of lactation, and their putative associations with maternal–infant dyad metadata, has the potential to provide insights into mechanisms of milk-component production and regulation, as well as variability between individuals (1). We confirm that the majority of cells in human breast milk are epithelial cells, specifically lactocytes, and that cell-type frequencies are dynamic over the course of lactation. Analysis of lactocytes reveals a continuum of cell states characterized by subtle transcriptional changes that point to changing populations of milk component-producing epithelial cells whose activities may be hormonally regulated. We also identify several subclusters of macrophages in hBM that are enriched for tolerogenic functions. Taken together, our data provide a detailed longitudinal study of breast milk cells with single-cell resolution. Further understanding of cells over the course of lactation—including B cells, macrophages, and LC1 cells—will build knowledge of the role of breast milk in infant development by identifying: 1) cells that are transferred to infant gut, 2) the molecules they produce that are important for gut and immune system development, and 3) the nutrients supplied in hBM (7, 72). Improved understanding of pathways and activities of breast milk-producing cells will further inform lactation health and could provide baseline information for studies of adverse lactation outcomes. Furthermore, it will aid in establishing eligibility criteria for milk bank donation, potentially allowing donors to contribute milk after the typical 1-y postpartum limit (73).

## Materials and Methods

### Donor Enrollment and Breast Milk Collection.

Donors were enrolled in the Massachusetts Institute of Technology (MIT) Milk Study under an approved protocol (Protocol #1811606982) and samples were de-identified prior to use in the study. Donors were recruited at hospitals, research institutes, and clinics around the Boston, Massachusetts, area, primarily on the MIT campus. Donors expressed milk using their method of choice and, where possible, provided that information in questionnaires for each sample. To minimize diurnal variations in cell composition, donors provided milk in the mornings between 6:00 AM and 9:00 AM (74, 75). We also collected extensive donor-supplied metadata for each sample (Dataset S1), including information about maternal and infant health. Donors collected a minimum of 0.5 mL of milk, placed in study-provided sample collection bags, and kept on ice until the sample was collected. Samples were processed as close to expression as possible (up to 6 h) and kept on ice until cells were isolated. Donors also provided answers to the study questionnaire with each sample. Donors provided milk at various time points, covering the following milk stages: early 3 to 6 d postpartum (colostrum/early), transitional (10 to 14 d), mature (15 to 18 d), and several later stages (late 1: 5 to 13 wk; late 2: 14 to 25 wk; late 3: 26 to 33 wk; and late 4: 34 to 90 wk). Breast milk was sampled from 15 mothers between the ages of 25 to 34 y (median age 31 y). All pregnancies were full term with seven donors reporting induced labor, four reporting C-sections, and all but two donors reporting no prior pregnancies. Four donors began hormonal birth control during the sampling period. Eight total samples from six donors were collected after starting day care.

### Cell Isolation.

To isolate cells directly from whole milk, samples were processed as previously described (39). Briefly, milk was diluted 1:1 with cold PBS and cells were pelleted by centrifugation for 10 min at 350 × *g.* After removal of skim milk and the fat layer, cells were transferred to a clean tube in 1 mL of cold PBS and washed three times in 10 mL of cold PBS. The final cell pellet was resuspended in 1 mL of cold complete RPMI media (ThermoFisher) containing 10% FBS and 5% pen/strep (ThermoFisher). Cells were counted with a hemocytometer and Seq-Well S^3^ was performed, as described below (76). For experiments comparing milk-handling and cell-isolation methods, cells were isolated as described above from milk that had been sorted at 4 °C or at −20 °C overnight. Frozen milk was thawed in a 37 °C water bath prior to cell isolation. For sorting of live cells, milk cells were isolated directly from milk and stained according to the manufacturers protocol for Calcein violet (ThermoFisher) and Sytox green (Invitrogen) prior to sorting for Calcein violet^+^ and Sytox green^−^ cells on a Sony Sorter (SH800S). For enrichment of live cells, directly isolated milk cells were processed according to the manufacturer’s instructions (EasySep Dead Cell Removal [Annexin V] Kit).

### Generation of scRNA-seq Data with Seq-Well S^3^.

Seq-Well S^3^ was performed as described previously (76, 77). For each milk sample, about 15,000 cells were loaded onto each array preloaded with uniquely barcoded mRNA capture beads (ChemGenes). Arrays were washed with protein-free RPMI media, then sealed with polycarbonate membranes. Arrays were incubated at 37 °C for 30 min to allow membranes to seal, then transferred through a series of buffer exchanges to allow for cell lysis, transcript hybridization, bead washing, and bead recovery from arrays postmembrane removal. Reverse transcription was performed with Maxima H Minus Reverse Transcriptase (ThermoFisher), excess primers were removed using an Exonuclease I digestion (New England Biolabs), second-strand synthesis was performed, and whole-transcriptome amplification by PCR was performed using KAPA Hifi PCR Mastermix (Kapa Biosystems). Whole-transcriptome amplification product was purified using Agencourt Ampure beads (Beckman Coulter) and dual-indexed 3′ digital gene-expression sequencing libraries were prepared using Nextera XT (Illumina). Libraries were sequenced on a NovaseqS4 or NovaseqS2 with a paired-end read structure (R1: 20 bases; I1: 8 bases; I2: 8 bases; R2: 50 bases) and custom sequencing primers.

### Analysis of scRNA-seq Data.

#### Alignment and quality control.

Data were aligned using the Dropseq-tools pipeline on Terra (https://app.terra.bio/) to human reference genome hg19. Sequencing saturation curves were generated using custom scripts to ensure adequate sequencing depth.

#### Clustering and cell identification.

Samples were split into milk-stage groups for initial clustering and doublet identification. For each sample, Scrublet was run with default parameters and cells identified as doublets were removed from downstream analysis (78). For each milk stage, all samples were combined into a single Scanpy object, cells were filtered with parameters: >400 genes, >750 UMI, <750 counts, <20% UMIs from mitochondrial genes. UMI counts were log-normalized and the top 2,000 variable genes were identified with the batch_key parameter set to “sample.” PC analysis was run on scaled data, and a nearest neighbor map was calculated with 15 neighbors and 25 principal components (PCs prior to running Uniform Manifold Approximation and Projection (UMAP) for visualization. Resulting clusters were robust to multiple choices of clustering parameters. Clustering of resulting transcriptomes was performed using Leiden clustering in the Scanpy (https://scanpy.readthedocs.io/en/stable/) package independently on samples of each milk stage (79). Clusters were classified as immune cells or epithelial cells for further subclustering based on expression of *PTRPC* (immune cells) and *LALBA* (epithelial cells). Upon subclustering on each of these subsets, doublets were identified as clusters coexpressing multiple lineage markers and were removed. Subclustering was performed on the applicable clusters from all time points combined, as reviewed previously (80).

#### Pseudobulk marker gene identification.

To identify marker genes for cell-type clusters whose specificity to Leiden clusters or cell subgroups was consistent across donors and samples, we utilized pseudobulk marker gene identification (81–83). Raw gene-expression counts were pooled by sample and cluster such that one pseudobulk population was created for each cluster found in each sample. Psuedobulk groups were filtered to include only sample-subcluster pairs containing at least 10 cells. Differential expression between clusters of one cell type and all other clusters was executed using a Wald test in DESeq2 with the design formula “∼donor + is.thiscelltype”, where the factor ‘is.thiscelltype’ is set to TRUE for pseudobulk populations from the cluster of interest and FALSE for other clusters (84). These pseudobulk marker genes were filtered for adjusted *P* < 0.05, percent expression of single cells in the cluster >30%, and DESeq2-calculated log_2_ fold-change > 0.4. Pseudobulk marker genes of all cell types (Dataset S2) and epithelial cell groups (Dataset S6), and top marker genes sorted by difference in percent of cells expressing in-cluster compared to out-of-cluster, are visualized in Figs. 1*C* and 3*D*, respectively.

#### Epithelial cell subclustering.

Epithelial subclustering was performed on combined cells from all samples to identify major cell states within the data and characterize their changes in gene expression over the course of lactation. To enable these analyses, we identified cell groups that were either distinct enough to be robust to clustering parameter selection or, for groups of cells whose core identifying gene-expression profiles could not be defined with respect to other clusters, similar clusters were merged and further analysis identified genes changing over time. Subclustering proceeded by rediscovering the top 3,000 variable genes on the epithelial subset, rerunning PC analysis on these genes, and clustering with Leiden clustering with resolution 0.7 and 10 neighbors on 22 PCs (*SI Appendix*, Fig. S8 *A* and *B*). See *SI Appendix*, *SI Text* for additional information on these cluster assignments. After clustering and merging, we represented the diversity of epithelial cells within each sample using Shannon entropy implemented with the Python function scipy.stats.entropy (*SI Appendix*, Fig. S8*K*).

#### Immune cell subclustering.

Immune cells were subclustered separately and refiltered to remove additional doublets. To accomplish the latter, immune cells were clustered with a known subset of secretory epithelial cells from our epithelial cell data. This allowed us to generate a gene signature derived of PC1-specific genes to define lactocytes or monocytes with high confidence (Dataset S5). We performed module scoring with these in R (v3.6.2) with Seurat (V3), allowing us to stringently filter for immune cells that scored highly for lactocyte gene expression (>2.5 SDs above the mean lactocyte module score) (85). Finally, we identified any additional doublets based on dual expression of key lineage markers as described above. We performed subclustering analyses by renormalizing the data, finding the top 2,000 variable genes, rescaling the data, running PC analysis, then performing additional UMAP visualization with the first 15 PCs. Supervised marker gene identification was performed across cell types using Seurat’s Wilcoxon rank-sum test. We also performed subclustering analyses on the monocytes and macrophages as these were the most abundant immune cell type. These cells were renormalized, the top 2,000 variable genes were identified, and the data were clustered across several resolutions to identify resolutions that produced nonredundant clusters (resolution = 0.2), as determined by marker-gene identification using Seurat’s Wilcoxon rank-sum test. Analysis of clustering robustness by leave-one-donor-out clustering described in *SI Appendix*, *Supplementary Methods*.

#### Identification of time-varying genes.

Time-associated genes were identified for each cluster using pseudobulk analysis. First, the raw counts of all cells in each sample in each cluster were summed to create sample- and cluster-specific pseudobulk data. Then DESeq2 was used to identify genes varying over the course of lactation in each subcluster using a likelihood ratio test between the design formula “∼ 0 + donor + days_postpartum” over “∼0 + donor” (84). Samples with a minimum of 10 cells in a cluster were included in the analysis, and samples from more than 400 d postpartum were excluded from time series analyses to avoid the small number of very late samples driving a disproportionate amount of variation due to the large gap in time between samples before 400 d postpartum and after. Genes with in-cluster single-cell percent expression >20% and adjusted *P* < 0.05 were included in downstream visualization and enrichment analyses. Heatmaps represent row *z*-scored, log-normalized per sample expression of genes of interest. PC analysis on pseudobulk samples from each epithelial subset was used to identify the primary axis of variation within each subset by identifying the sample metadata and genes correlated with the first PC. The first PC of the LC1 epithelial and secretory lactocyte subsets was highly correlated with time postpartum, so time-dependent gene analyses were focused on these subsets (Dataset S7). We classified universal epithelial cell time-varying genes as genes associated with time and changing in the same direction in both LC1 epithelial and secretory epithelial subsets (Dataset S8). Time-varying genes in opposite directions in the LC1 epithelial and secretory epithelial subsets were also identified (Dataset S8).

#### Identification of metadata-associated cellular populations.

Associations between collected covariates and cellular population proportions were tested using generalized additive models. For each sample, cell cluster proportions were calculated from the numbers of cells found in each broad cell type by dividing the number of cells in that cluster by the total cells in that sample. Then a generalized additive model was run for each cell type on samples collected earlier than 400 d postpartum using the mgcv R package with model formula “celltype_proportion ∼ donor + s(time_postpartum_days, *k* = 7)” (86). Additional covariates, including daycare attendance, infant illness, breast soreness, supplementation with formula, use of hormonal birth control, solid food consumption, and recent vaccinations were tested with model formulas following the pattern “celltype_proportion ∼ donor + <covariate>”. Only samples with complete metadata for a given covariate were included in the corresponding comparison (*SI Appendix*, Table S2). In place of multiple testing correction, a conservative *P* value threshold of *P* < 0.005 was used. Because it is possible that changes in epithelial cell composition and immune cell composition might be unrelated, associations with time and other metadata factors were also tested on proportional abundances within each of these subsets, as described above. Full model results are shown in *SI Appendix*, Table S2.

#### Functional enrichment analysis on epithelial cells.

Functional enrichment analysis on top marker genes was performed using Enrichr using the gseapy package with the gene set GO_Biological_Processes_2021 (87, 88). GO terms were curated to identify a limited informative set of terms (*SI Appendix*, *Supplementary Methods*). Heatmap visualizations display per subset mean gene set score for all genes in the GO term *z*-scored across subsets. A similar process identified GO terms changing over time postpartum. GO terms identified to be changing in the same direction in both the LC1 epithelial and secretory lactocyte clusters were considered epithelial cell-wide time-varying processes. Full results are in Dataset S9.

## Supplementary Material

Supplementary File

Supplementary File

Supplementary File

Supplementary File

Supplementary File

Supplementary File

Supplementary File

Supplementary File

Supplementary File

Supplementary File

## Data Availability

Notebooks to reproduce all analyses performed in R and Python are available for download (https://github.com/ShalekLab/MIT_Milk_Study) (89). Raw data are available at the Data Use and Oversight System controlled access repository https://duos.broadinstitute.org/ (accession no. DUOS-000140) (90). Aligned count data and annotations can be downloaded and explored as part of The Alexandria Project on Single Cell Portal (https://singlecell.broadinstitute.org/single_cell/study/SCP1671) (91).
