# Supplementary material for: Cellular and transcriptional diversity over the course of human lactation
Source: Proc Natl Acad Sci U S A. 2022 Apr 4;119(15):e2121720119. doi: 10.1073/pnas.2121720119 (PMC9169737; doi:10.1073/pnas.2121720119)
Supplement: Supplementary File [file pnas.2121720119.sapp.pdf]

PNAS
www.pnas.org

Supplementary Information for
Cellular and transcriptional diversity over the course of human lactation

Sarah K. Nyquist[1,2,3,4], Patricia Gao[3], Tessa K. J. Haining[3], Michael R. Retchin[3], Yarden Golan[5], Riley S. Drake[1,3,6], Kellie Kolb[1,3], Benjamin E. Mead[1,3], Nadav Ahituv[5], Micaela E. Martinez[7], Alex K. Shalek[1,2,3,6,8,9,10+], Bonnie Berger[1,4+], Brittany A. Goods[11,+]

[1]Broad Institute of MIT and Harvard, Cambridge, MA, 02142

[2]Program in Computational and Systems Biology, Massachusetts Institute of Technology, Cambridge, MA 02139

[3]Department of Chemistry and Institute for Medical Engineering & Science, Massachusetts Institute of Technology, Cambridge, MA 02139

[4] Computer Science and Artificial Intelligence Laboratory and Department of Mathematics, MIT, Cambridge, Massachusetts, 02139

[5]Department of Bioengineering and Therapeutic Sciences and Institute for Human Genetics UCSF, University of California San Francisco, San Francisco, CA 94143

[6]Ragon Institute of MGH, MIT, and Harvard, Cambridge, MA 02139

[7]Department of Biology, Emory University, Atlanta, GA 30322

[8]Koch Institute for Integrative Cancer Research, Massachusetts Institute of Technology, Cambridge, MA 02139

[9]Division of Health Science & Technology, Harvard Medical School, Boston, MA 02115

[10]Department of Immunology, Massachusetts General Hospital, Boston, MA 02114

[11]Thayer School of Engineering and Program in Quantitative Biomedical Sciences, Dartmouth College, Hanover, NH 03755

[+]equal

*Bonnie Berger, Brittany A. Goods, Alex K. Shalek

This PDF file includes:

Supplementary text
Figures S1 to S6
Tables S1, S2
Legends for Datasets S1 to S9
SI References

Other supplementary materials for this manuscript include the following:

Datasets S1 to S9

Supplementary Information Text

Supplementary Methods

Epithelial cell clustering

Our goals in describing the epithelial cells are to 1) identify distinct functional subclusters involved in different roles during lactation, 2) ask if the proportional abundance of any of these subclusters changed over the course of lactation, and 3) identify any transcriptional changes within any subcluster over the course of lactation which might facilitate an understanding of the functional shifts in these subclusters.

To choose the representation of the data that best answers those questions, multiple Leiden clustering parameters were considered, and the resolution was selected to maximize the number of clusters with distinct marker genes and enriched GO terms and reduce the amount of post-clustering merging required to identify these distinct genes (Fig. S8A,B,J, S9). We note that within the large LC2 celltype, several cluster boundaries were relatively stable at multiple clustering resolutions, but several shifted inconsistently at each resolution, grouping different subsets of cells together (Fig. S7A). Cells in clusters 0, 1, 2, and 3 were unstable across clustering parameters, so these clusters were merged into the secretory lactocyte cluster due to shared expression of various canonical lactation-related genes (*CHRDL2, FASN CEL)* with few other discriminating markers (Fig. S8G,J). These four clusters changed in abundance over time (milk stages) (Fig. S8E), but because of the lack of discriminating markers, we determined that inference of functional changes over time would be better facilitated with direct regression of gene expression over time across all four clusters instead of by attempting to describe four different subclusters of secretory lactocytes which changed in abundance over time. Despite many shared functions with clusters 0, 1, 2, and 3, cluster 5 was left as its own cluster due to high mitochondrial gene percentage and its consistent separation across clustering resolutions (Fig. S8H,I). Clusters 9, 6 and 8 shared a distinct transcriptional signature and were merged into the LC1 cluster. Clusters 4 and 11 were merged into a single *KRT* high 1 cluster due to cluster 11's specificity to a single donor, and cluster 7 remained as a single *KRT* high 2 cluster. Additionally, these clusters each contained cells from multiple donors and were robust to leave-one-donor-out clustering (Fig. S8F).

Leave one donor out clustering of macrophage cells

Because some clusters were dominated by individual donors, we sought to ensure that single no single donor was driving the biological conclusions from our clustering results by preforming leave-one-donor-out clustering analysis on the macrophage subset. We repeated the variable gene identification (top 2,000) and clustering at resolution=0.2, as was done on the full macrophage dataset, on the macrophage cells with each donor excluded. We compared clustering assignments of the remaining cells to the clusters identified on all donors using the Adjusted Rand Index, a metric which measures the similarity between two cluster assignments ignoring permutations where a score of 0 indicates a random permutation and a score of 1.0 indicates an exact match. We found that leaving out any donor except donor BM05 resulted in an adjusted Rand index of 0.9-0.98 indicating a high consensus regardless of the inclusion of an individual donor. We further examined the clustering that excluded donor BM05 further to understand the impact of including this donor on the results. BM05 is the donor with the highest number of cells in the macrophage cluster, most of which were assigned to sub-cluster 0 (Fig. S6A). To ensure that no biological conclusions depended on the inclusion of this donor, we repeated the marker gene identification (Fig. S6B) with this donor excluded as well as comparisons of M1 and M2 scores across the 5 clusters identified (Fig. S6C) and found no differences which would impact the biological interpretation of the results.

GO Term reduction for functional enrichment of epithelial cells

Functional enrichment analysis on top epithelial cell subcluster marker genes was performed using Enrichr using the gseapy package with the gene set GO_Biological_Processes_2021 (1,2). Due to the hierarchical structure of the GO database and the overlapping functions of many of the marker genes of the epithelial cell subclusters, representative GO terms were identified through a series of filtering and curating steps. For each subcluster, significantly enriched terms were grouped based on shared marker genes found to be overlapping with the GO term. These grouped terms were further grouped between subclusters based on shared term ID or shared genes. The mean gene set score was calculated for each epithelial cell group and enriched GO term using the Scanpy function “score_genes” (3). For each group of GO terms, the terms with the highest variance of mean gene scores across epithelial subgroup was chosen such that each epithelial subgroup had between 7 and 15 GO terms for which they had the maximum mean gene score. To avoid redundant terms, GO terms were also merged based on high overlap of genes in the full reference GO term gene list.

Time-dependent enriched GO terms were identified for genes positively and negatively associated with time postpartum separately and for both LC1 epithelial and secretory lactocyte clusters. These GO terms were similarly curated with an additional filtering step of correlation of the gene set scores over time postpartum in the same direction as the set of differential genes used (e.g. positive correlation for GO terms enriched in the gene list increasing with time).

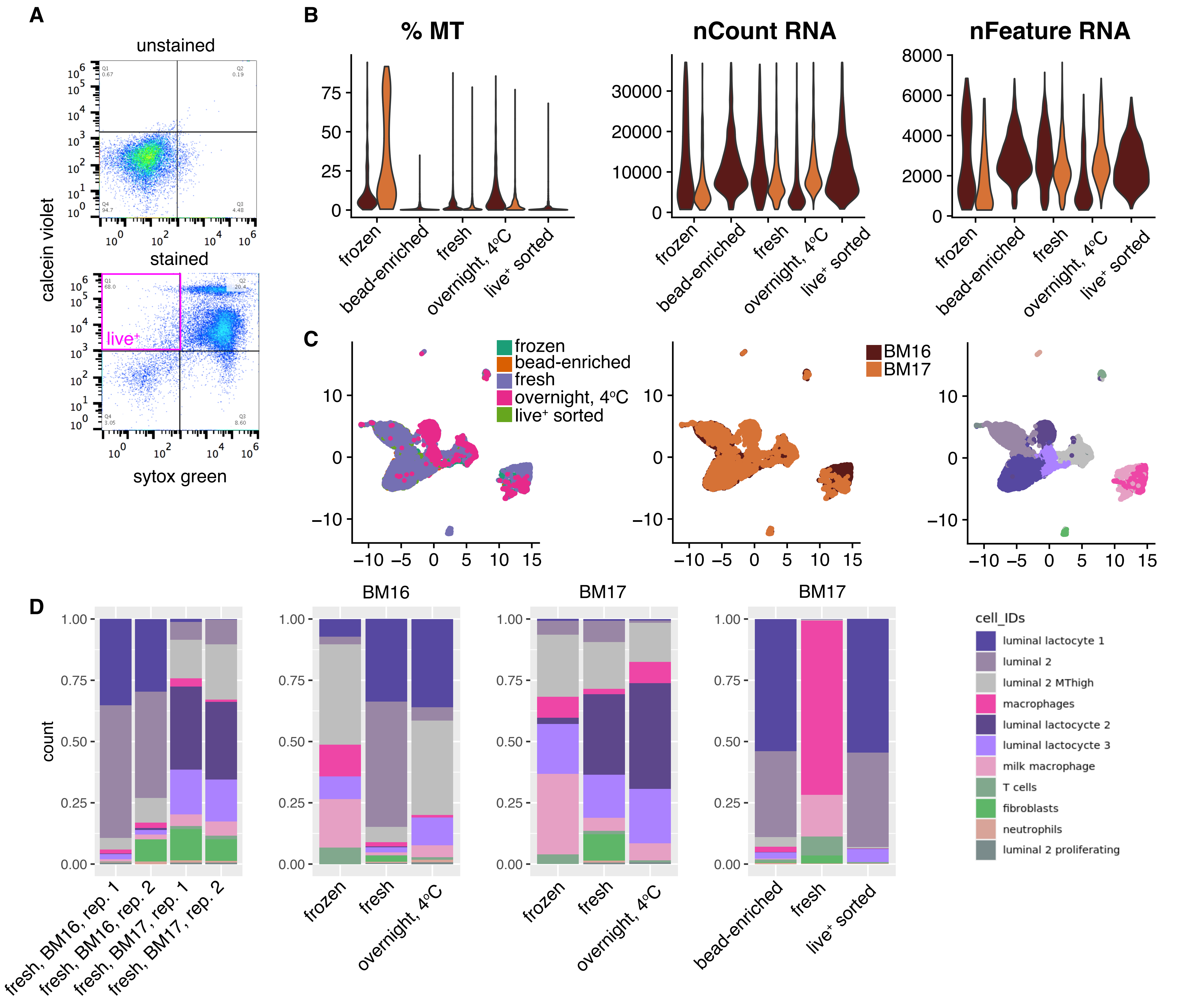
A
unstained
stained
live+
calcein violet
sytox green
B
% MT
nCount RNA
nFeature RNA
frozen
bead-enriched
fresh
overnight, 4°C
live+ sorted
C
frozen
bead-enriched
fresh
overnight, 4°C
live+ sorted
BM16
BM17
D
BM16
BM17
BM17
count
fresh, BM16, rep. 1
fresh, BM16, rep. 2
fresh, BM17, rep. 1
fresh, BM17, rep. 2
cell_IDs
luminal lactocyte 1
luminal 2
luminal 2 MThigh
macrophages
luminal lactocyte 2
luminal lactocyte 3
milk macrophage
T cells
fibroblasts
neutrophils
luminal 2 proliferating

Fig. S1. Comparison of cell isolation methods from human breast milk. A. Representative flow sorting plot for live cells (calcein violet+sytox-). **B.** Violin plots of quality control metrics (%MT, nCount RNA, nFeature RNA) for each processing condition across two donors. **C.** UMAPs of combined data colored by processing method, donor, and cell identity. **D.** Stacked bar charts showing relative frequencies of cell types for each indicated condition.

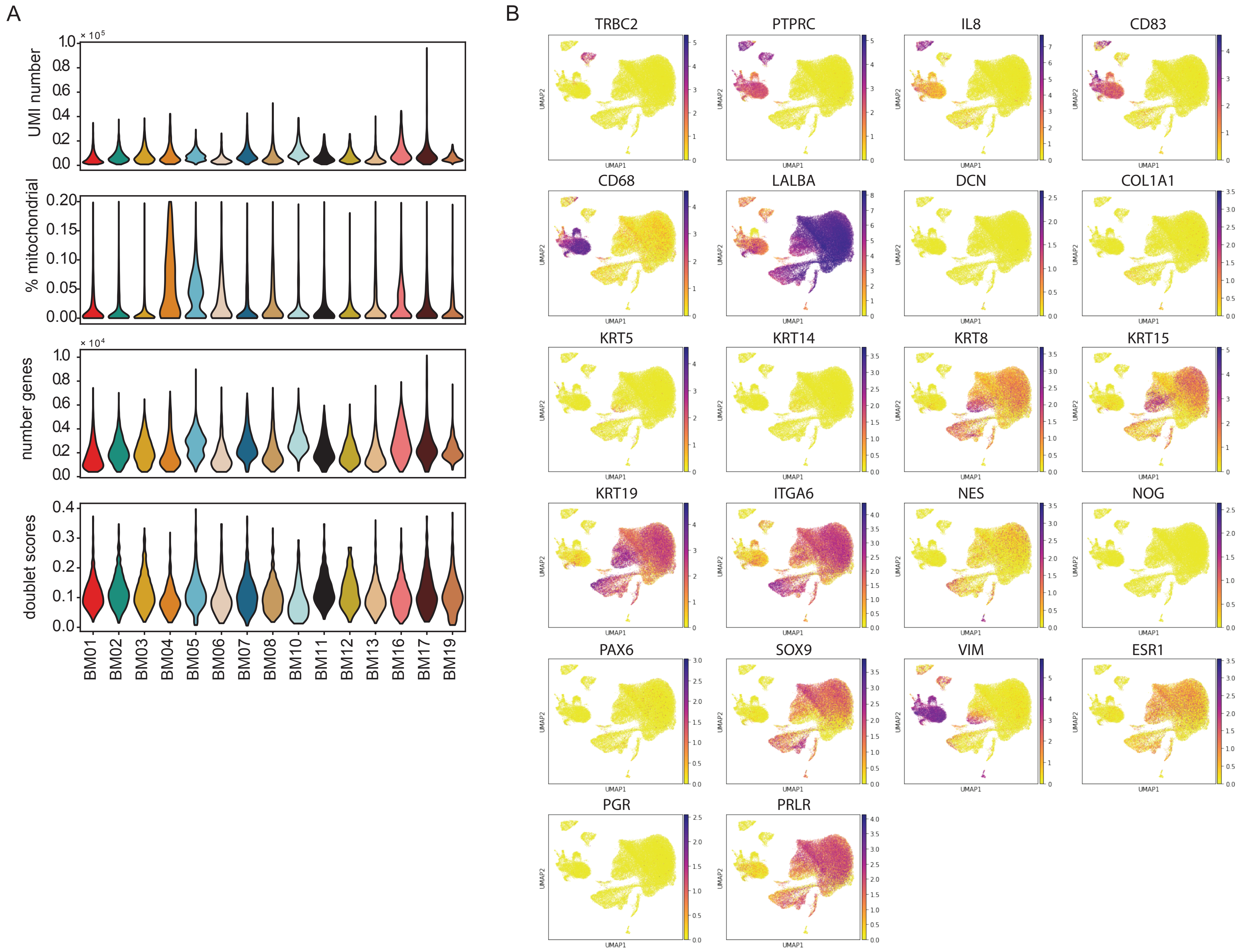

A
B
UMI number
% mitochondrial
number genes
doublet scores
× 10^5
× 10^4
BM01
BM02
BM03
BM04
BM05
BM06
BM07
BM08
BM10
BM11
BM12
BM13
BM16
BM17
BM19
TRBC2
PTPRC
IL8
CD83
CD68
LALBA
DCN
COL1A1
KRT5
KRT14
KRT8
KRT15
KRT19
ITGA6
NES
NOG
PAX6
SOX9
VIM
ESR1
PGR
PRLR
UMAP1
UMAP2

Fig. S2. Sequencing quality control and feature plots. **A**. Violin plots of quality control metrics for each donor in the cohort. **B**. Feature plots showing expression of select genes.

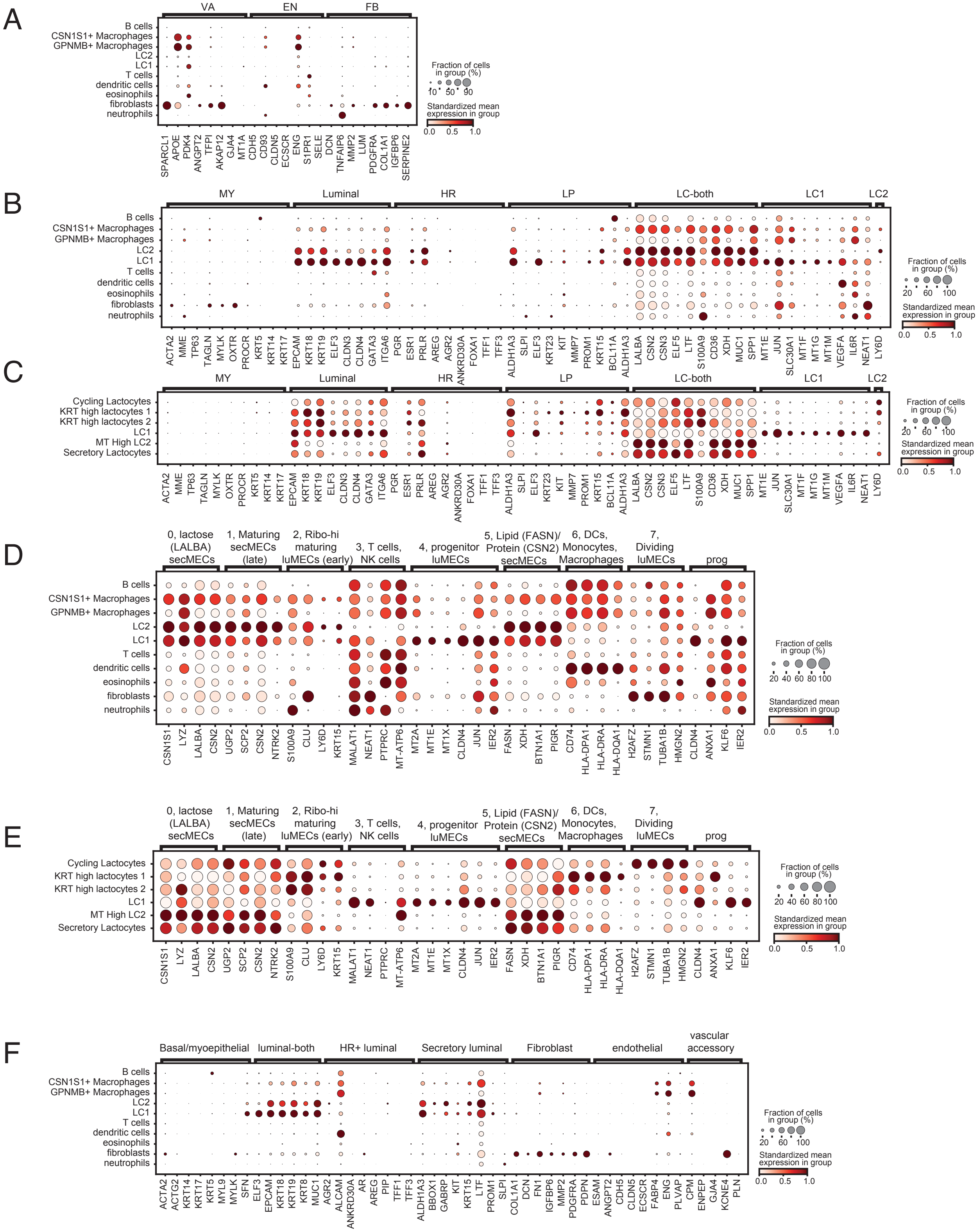
A
VA
EN
FB
B cells
CSN1S1+ Macrophages
GPNMB+ Macrophages
LC2
LC1
T cells
dendritic cells
eosinophils
fibroblasts
neutrophils
SPARCL1 APOE PDK4 ANGPT2 TFPI AKAP12 GJA4 MT1A CDH5 CD93 CLDN5 ECSCR ENG S1PR1 SELE DCN TNFAIP6 MMP2 LUM PDGFRA COL1A1 IGFBP6 SERPINE2
Fraction of cells in group (%)
10 50 90
Standardized mean expression in group
0.0 0.5 1.0
B
MY
Luminal
HR
LP
LC-both
LC1
LC2
ACTA2 MME TP63 TAGLN MYLK OXTR PROCR KRT5 KRT14 KRT17 EPCAM KRT18 KRT19 ELF3 CLDN3 CLDN4 GATA3 ITGA6 PGR ESR1 PRLR AREG AGR2 ANKRD30A FOXA1 TFF1 TFF3 ALDH1A3 SLPI ELF3 KRT23 KIT MMP7 PROM1 KRT15 BCL11A ALDH1A3 LALBA CSN2 CSN3 ELF5 LTF S100A9 CD36 XDH MUC1 SPP1 MT1E JUN SLC30A1 MT1F MT1G MT1M VEGFA IL6R NEAT1 LY6D
Fraction of cells in group (%)
20 60 100
Standardized mean expression in group
0.0 0.5 1.0
C
Cycling Lactocytes
KRT high lactocytes 1
KRT high lactocytes 2
LC1
MT High LC2
Secretory Lactocytes
D
0, lactose (LALBA) secMECs
1, Maturing secMECs (late)
2, Ribo-hi maturing luMECs (early)
3, T cells, NK cells
4, progenitor luMECs
5, Lipid (FASN)/ Protein (CSN2) secMECs
6, DCs, Monocytes, Macrophages
7, Dividing luMECs
prog
CSN1S1 LYZ LALBA CSN2 UGP2 SCP2 CSN2 NTRK2 S100A9 CLU LY6D KRT15 MALAT1 NEAT1 PTPRC MT-ATP6 MT2A MT1E MT1X CLDN4 JUN IER2 FASN XDH BTN1A1 PIGR CD74 HLA-DPA1 HLA-DRA HLA-DQA1 H2AFZ STMN1 TUBA1B HMGN2 CLDN4 ANXA1 KLF6 IER2
Fraction of cells in group (%)
20 40 60 80 100
E
F
Basal/myoepithelial
luminal-both
HR+ luminal
Secretory luminal
Fibroblast
endothelial
vascular accessory
ACTA2 ACTG2 KRT14 KRT17 KRT5 MYL9 MYLK SFN ELF3 EPCAM KRT18 KRT19 KRT8 MUC1 AGR2 ALCAM ANKRD30A AR AREG PIP TFF1 TFF3 ALDH1A3 BBOX1 GABRP KIT KRT15 LTF PROM1 SLPI COL1A1 DCN FN1 IGFBP6 MMP2 PDGFRA PDPN ESAM ANGPT2 CDH5 CLDN5 ECSCR FABP4 ENG PLVAP CPM ENPEP GJA4 KCNE4 PLN

Fig. S3: Comparisons to existing MEC dataset cluster marker genes: Each plot shows gene expression of cells and clusters from this study (rows) using marker gene and cluster labels from previous studies (columns) **A.** Dotplot of marker genes identified in stromal cell clusters in non-lactating breast tissue in (4) grouped by major celltype cluster. VA: vascular accessory, EN: endothelial, FB: fibroblast. **B.** Dotplot of marker genes identified in epithelial cell clusters in both non-lactating breast tissue and hBM in (4) grouped by major celltype cluster (**B**) and epithelial subcluster (**C**). **D.** Dotplot of marker genes for clusters identified in scRNA-Seq in hBM in (5) plotted across all major celltypes (**D**) and epithelial subclusters (**E**). **F.** Dotplot of marker genes identified in *EPCAM+* cells from non-lactating breast tissue in (6) plotted across major celltype clusters.

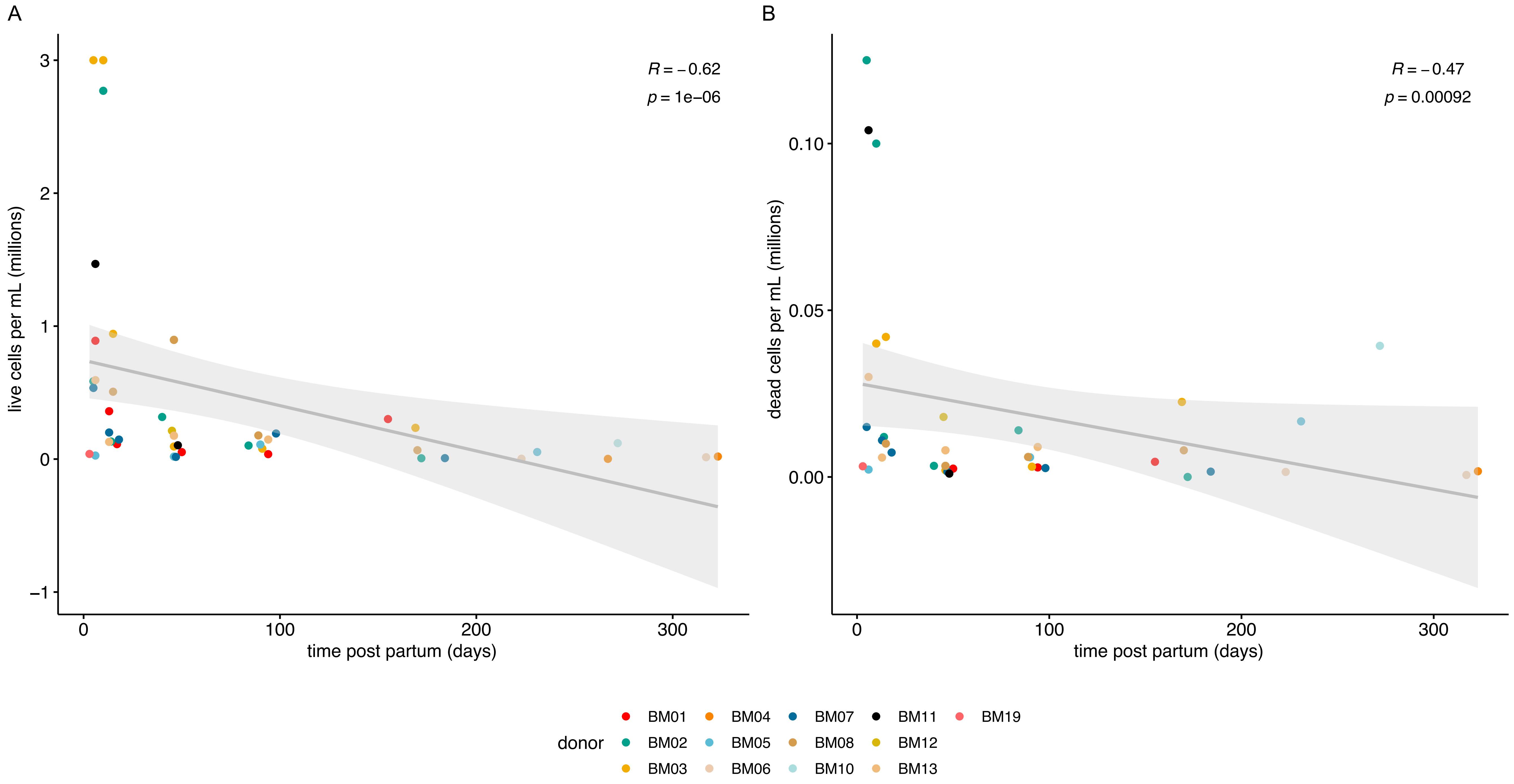
A
B
$R = -0.62$
$p = 1e-06$
$R = -0.47$
$p = 0.00092$
live cells per mL (millions)
dead cells per mL (millions)
time post partum (days)
time post partum (days)
3
2
1
0
−1
0.10
0.05
0.00
0
100
200
300
donor
BM01
BM02
BM03
BM04
BM05
BM06
BM07
BM08
BM10
BM11
BM12
BM13
BM19

Fig. S4. Cell counts over time in collected hBM samples. A. Live cells per mL and **B.** dead cells per mL by time postpartum in the cohort.

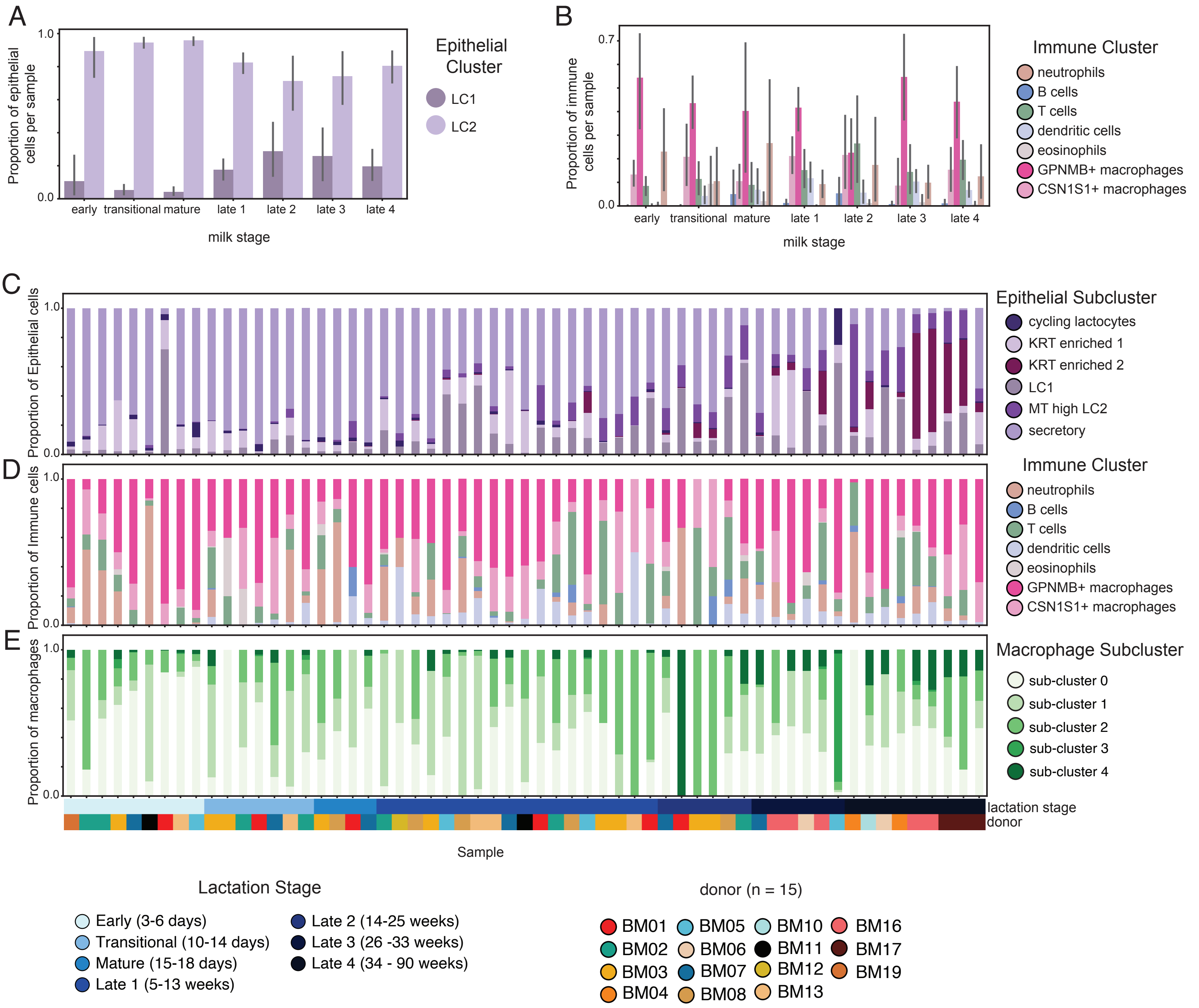
A
Proportion of epithelial cells per sample
1.0
0.0
early
transitional
mature
late 1
late 2
late 3
late 4
milk stage
Epithelial Cluster
LC1
LC2
B
Proportion of immune cells per sample
0.7
0.0
early
transitional
mature
late 1
late 2
late 3
late 4
milk stage
Immune Cluster
neutrophils
B cells
T cells
dendritic cells
eosinophils
GPNMB+ macrophages
CSN1S1+ macrophages
C
Proportion of Epithelial cells
1.0
0.0
Epithelial Subcluster
cycling lactocytes
KRT enriched 1
KRT enriched 2
LC1
MT high LC2
secretory
D
Proportion of Immune cells
1.0
0.0
Immune Cluster
neutrophils
B cells
T cells
dendritic cells
eosinophils
GPNMB+ macrophages
CSN1S1+ macrophages
E
Proportion of macrophages
1.0
0.0
Macrophage Subcluster
sub-cluster 0
sub-cluster 1
sub-cluster 2
sub-cluster 3
sub-cluster 4
lactation stage
donor
Sample
Lactation Stage
Early (3-6 days)
Transitional (10-14 days)
Mature (15-18 days)
Late 1 (5-13 weeks)
Late 2 (14-25 weeks)
Late 3 (26 -33 weeks)
Late 4 (34 - 90 weeks)
donor (n = 15)
BM01
BM02
BM03
BM04
BM05
BM06
BM07
BM08
BM10
BM11
BM12
BM13
BM16
BM17
BM19

Fig. S5: Celltype subset compositions across time and samples. A,B. Proportions of each major epithelial cluster (**A**) and immune cluster (**B**) per sample, split by milk stage. Error bars show standard deviation. **C-E**. Frequency of cell types identified for each sample within epithelial subclusters (**C**), immune cell subclusters (**D**), and macrophage subclusters (**E**). Lactation stage and donor number are represented by colors below the stacked bar plots

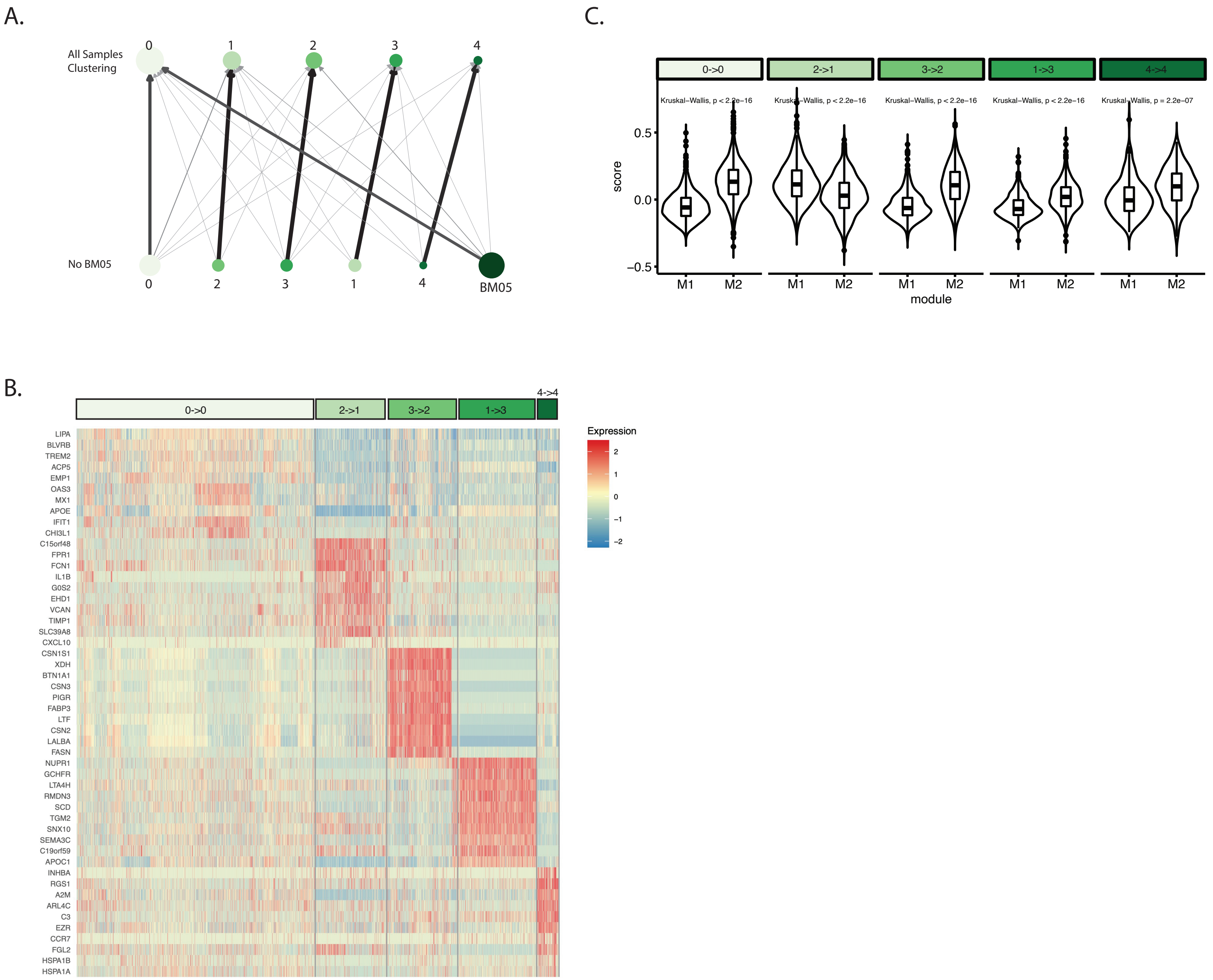
A.
All Samples Clustering
No BM05
0
1
2
3
4
BM05
C.
0->0
2->1
3->2
1->3
4->4
Kruskal–Wallis, p < 2.2e–16
Kruskal–Wallis, p = 2.2e–07
score
0.5
0.0
–0.5
M1
M2
module
B.
Expression
2
1
0
–1
–2
LIPA
BLVRB
TREM2
ACP5
EMP1
OAS3
MX1
APOE
IFIT1
CHI3L1
C15orf48
FPR1
FCN1
IL1B
G0S2
EHD1
VCAN
TIMP1
SLC39A8
CXCL10
CSN1S1
XDH
BTN1A1
CSN3
PIGR
FABP3
LTF
CSN2
LALBA
FASN
NUPR1
GCHFR
LTA4H
RMDN3
SCD
TGM2
SNX10
SEMA3C
C19orf59
APOC1
INHBA
RGS1
A2M
ARL4C
C3
EZR
CCR7
FGL2
HSPA1B
HSPA1A

Fig. S6: Macrophage sub-clustering with donor BM05 removed: A. Cell cluster reassignment by upon re-clustering without donor BM05. Circle sizes are proportional to proportion of cells in each cluster. Top row represents clusters computed on macrophages from all donors, bottom row represents clusters computed on macrophages from all donors except BM05 with cells from donor BM05 represented as the final circle. Edge weights represent the proportion of cells from BM05-excluded clusters which are assigned to each of the all-sample clusters. **B**. Top marker genes for each BM05-removed cluster, colored by the all-sample cluster which contains the highest proportion of those cells and for which the marker genes are most similar. Cell clusters labeled along the top of heatmap labeled with the format "BM05 removed cluster name"->"All samples cluster name". **C.** M1 and M2 module scores on cells from each BM05 removed clusters, labeled as in (**B**).

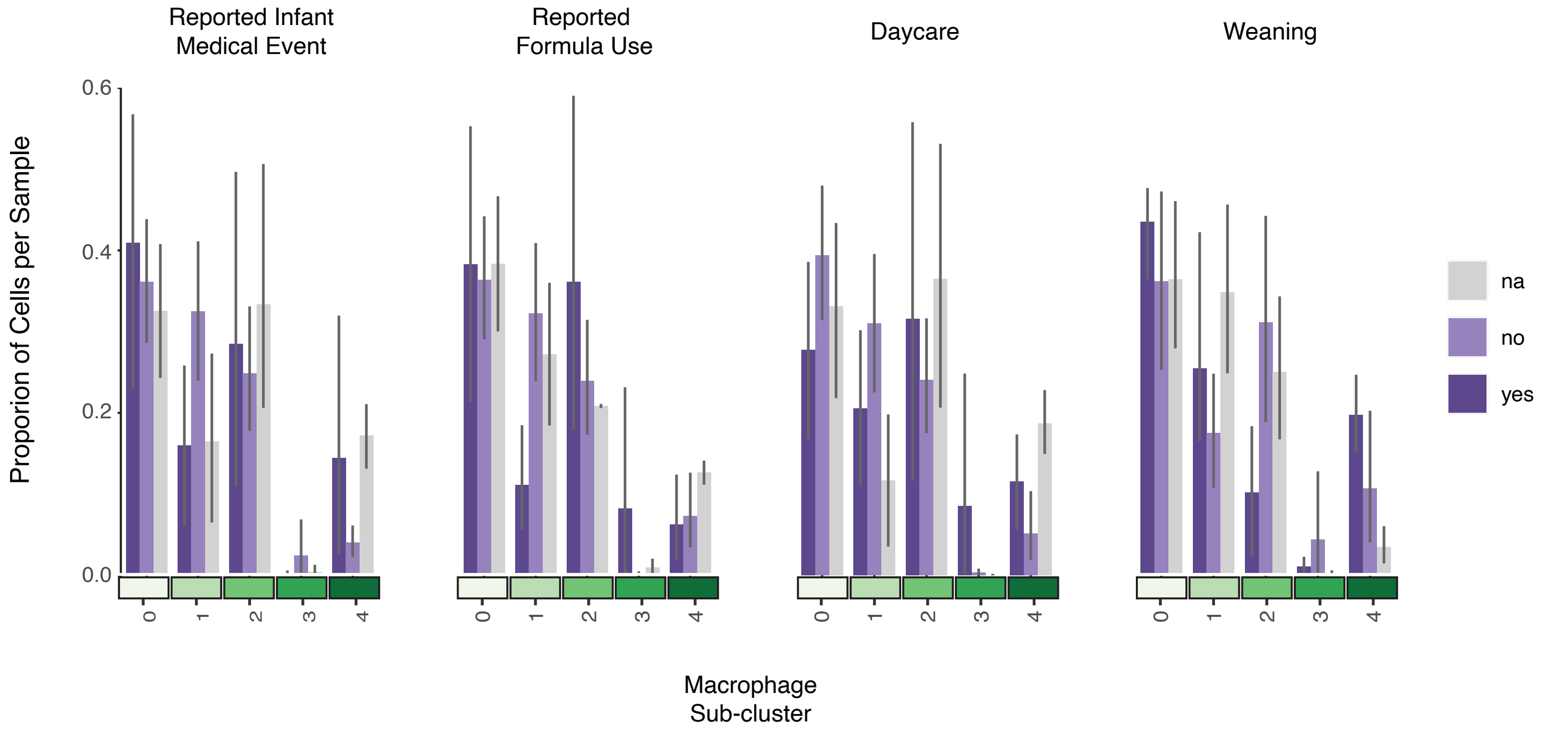
Reported Infant Medical Event
Reported Formula Use
Daycare
Weaning
Proporion of Cells per Sample
0.6
0.4
0.2
0.0
0
1
2
3
4
na
no
yes
Macrophage Sub-cluster

Fig. S7: Macrophage sub-cluster metadata comparisons: Composition of each sub-cluster as a function of infant medical events, formula use, daycare, weaning status.

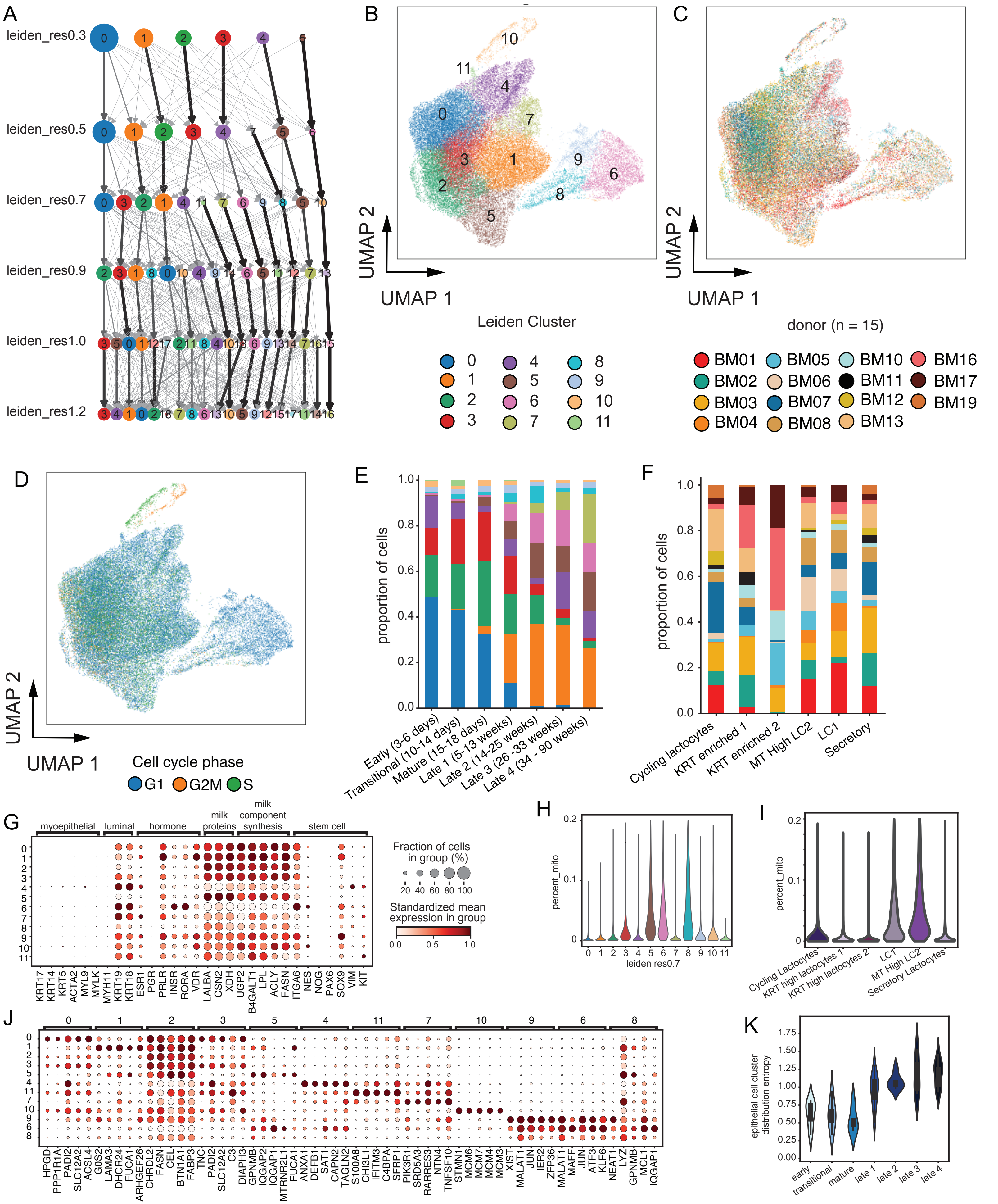
A
leiden_res0.3
leiden_res0.5
leiden_res0.7
leiden_res0.9
leiden_res1.0
leiden_res1.2
B
UMAP 1
UMAP 2
Leiden Cluster
C
UMAP 1
UMAP 2
donor (n = 15)
BM01 BM05 BM10 BM16
BM02 BM06 BM11 BM17
BM03 BM07 BM12 BM19
BM04 BM08 BM13
D
UMAP 1
UMAP 2
Cell cycle phase
G1 G2M S
E
proportion of cells
Early (3-6 days)
Transitional (10-14 days)
Mature (15-18 days)
Late 1 (5-13 weeks)
Late 2 (14-25 weeks)
Late 3 (26 -33 weeks)
Late 4 (34 - 90 weeks)
F
proportion of cells
Cycling lactocytes
KRT enriched 1
KRT enriched 2
MT High LC2
LC1
Secretory
G
myoepithelial
luminal
hormone
milk proteins
milk component synthesis
stem cell
Fraction of cells in group (%)
Standardized mean expression in group
KRT17 KRT14 KRT5 ACTA2 MYL9 MYLK MYH11 KRT19 KRT18 ESR1 PGR PRLR INSR RORA VDR LALBA CSN2 XDH UGP2 B4GALT1 LPL ACLY FASN ITGA6 NES NOG PAX6 SOX9 VIM KIT
H
percent_mito
leiden res0.7
I
percent_mito
Cycling Lactocytes
KRT high lactocytes 1
KRT high lactocytes 2
LC1
MT High LC2
Secretory Lactocytes
J
HPGD PPP1R1A PADI2 SLC12A2 ACSL4 G0S2 LAMA3 DHCR24 FUCA1 ARHGEF26 CHRDL2 FASN CEL BTN1A1 FABP3 TNC PADI2 SLC12A2 C3 DIAPH3 GPNMB IQGAP2 IQGAP1 MTRNR2L1 FUCA1 ANXA1 DEFB1 SAT1 CAPN2 TAGLN2 S100A8 CHI3L1 IFITM3 C4BPA SFRP1 PIK3R1 SRD5A3 RARRES3 NTN4 TNFSF10 STMN1 MCM6 MCM7 MCM4 MCM3 XIST MALAT1 JUN IER2 ZFP36 MALAT1 MAFF JUN ATF3 KLF6 NEAT1 LYZ GPNMB MCL1 IQGAP1
K
epithelial cell cluster distribution entropy
early
transitional
mature
late 1
late 2
late 3
late 4

Fig. S8. Epithelial sub-clustering A. Cell cluster reassignment of different Leiden clustering resolutions (labeled on left). Circle sizes are proportional to proportion of cells in each cluster. Edge weights and opacity represent the proportion of cells from lower clustering resolution which are assigned clusters in higher clustering resolution. **B.** UMAP visualizing epithelial cells colored by Leiden (resolution =0.7) clusters **C.** Donor **D**. and computationally defined cell cycle score. **E.** Proportion of cells during each milk stage colored by Leiden cluster. **F.** Proportion of cells in each epithelial sub-group originating from each donor. **G**. Mean expression in cell subset standardized within genes (color) and percent of cells expression (dot size) of canonical mammary epithelial marker genes and **J.** pseudobulk marker genes for each Leiden cluster **H.** Percent mitochondrial gene expression of each Leiden cluster and consensus cluster (**I**). **K.** Entropy of epithelial cell distribution per sample across each milk stage.

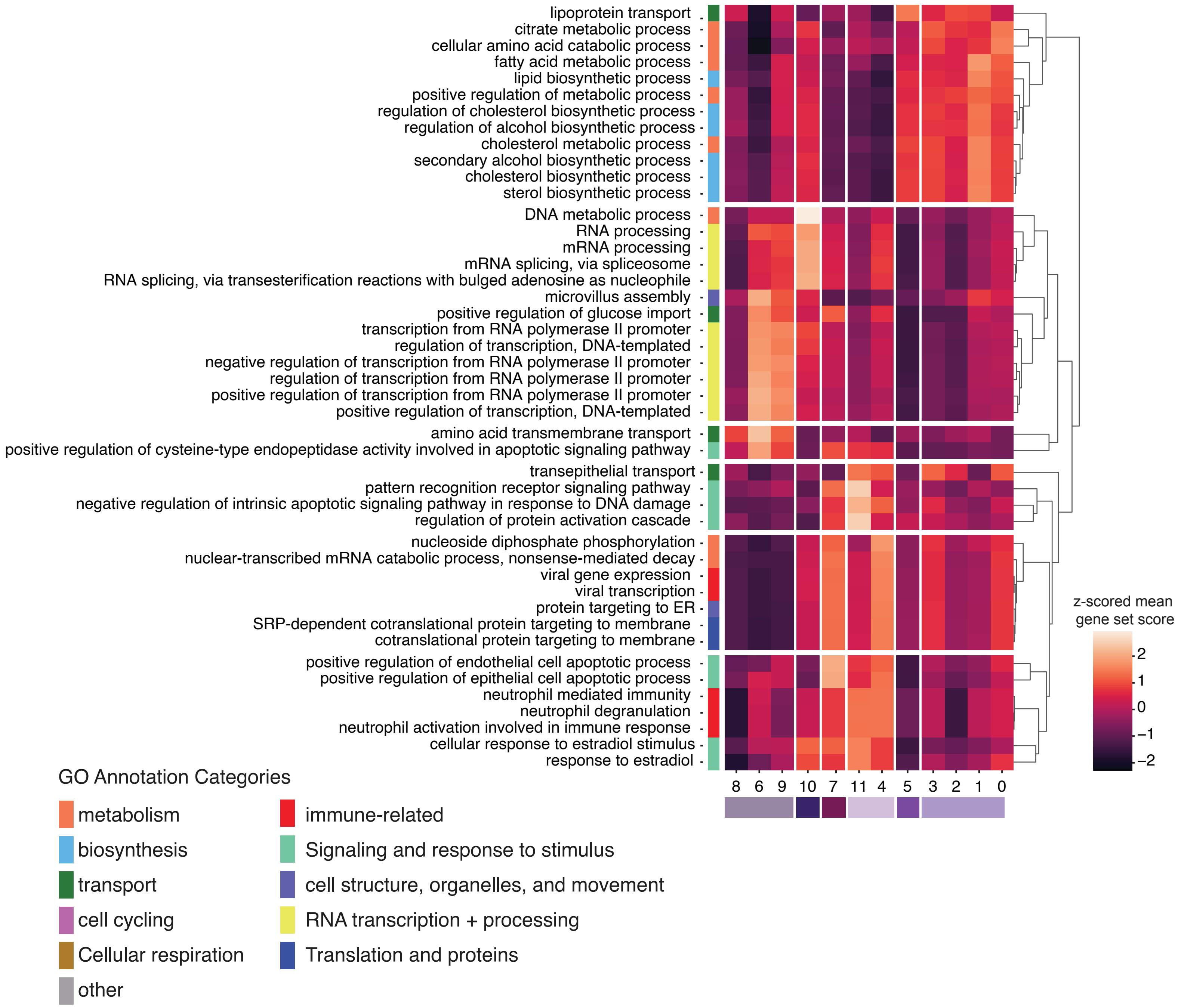
lipoprotein transport
citrate metabolic process
cellular amino acid catabolic process
fatty acid metabolic process
lipid biosynthetic process
positive regulation of metabolic process
regulation of cholesterol biosynthetic process
regulation of alcohol biosynthetic process
cholesterol metabolic process
secondary alcohol biosynthetic process
cholesterol biosynthetic process
sterol biosynthetic process
DNA metabolic process
RNA processing
mRNA processing
mRNA splicing, via spliceosome
RNA splicing, via transesterification reactions with bulged adenosine as nucleophile
microvillus assembly
positive regulation of glucose import
transcription from RNA polymerase II promoter
regulation of transcription, DNA-templated
negative regulation of transcription from RNA polymerase II promoter
regulation of transcription from RNA polymerase II promoter
positive regulation of transcription from RNA polymerase II promoter
positive regulation of transcription, DNA-templated
amino acid transmembrane transport
positive regulation of cysteine-type endopeptidase activity involved in apoptotic signaling pathway
transepithelial transport
pattern recognition receptor signaling pathway
negative regulation of intrinsic apoptotic signaling pathway in response to DNA damage
regulation of protein activation cascade
nucleoside diphosphate phosphorylation
nuclear-transcribed mRNA catabolic process, nonsense-mediated decay
viral gene expression
viral transcription
protein targeting to ER
SRP-dependent cotranslational protein targeting to membrane
cotranslational protein targeting to membrane
positive regulation of endothelial cell apoptotic process
positive regulation of epithelial cell apoptotic process
neutrophil mediated immunity
neutrophil degranulation
neutrophil activation involved in immune response
cellular response to estradiol stimulus
response to estradiol
8 6 9 10 7 11 4 5 3 2 1 0
z-scored mean gene set score
2
1
0
−1
−2
GO Annotation Categories
metabolism
biosynthesis
transport
cell cycling
Cellular respiration
other
immune-related
Signaling and response to stimulus
cell structure, organelles, and movement
RNA transcription + processing
Translation and proteins

Fig. S9. Epithelial sub-cluster GO heatmap. Reduced top Enrichr results from the gene ontology biological processes 2021 database on the marker genes for each Leiden clusters, colored by the mean gene set score for all genes in that pathway on cells in that subgroup, scaled by a z-score across subgroups. Leiden clusters ordered by the sub-group which they were merged into with colors corresponding to sub-group identity.

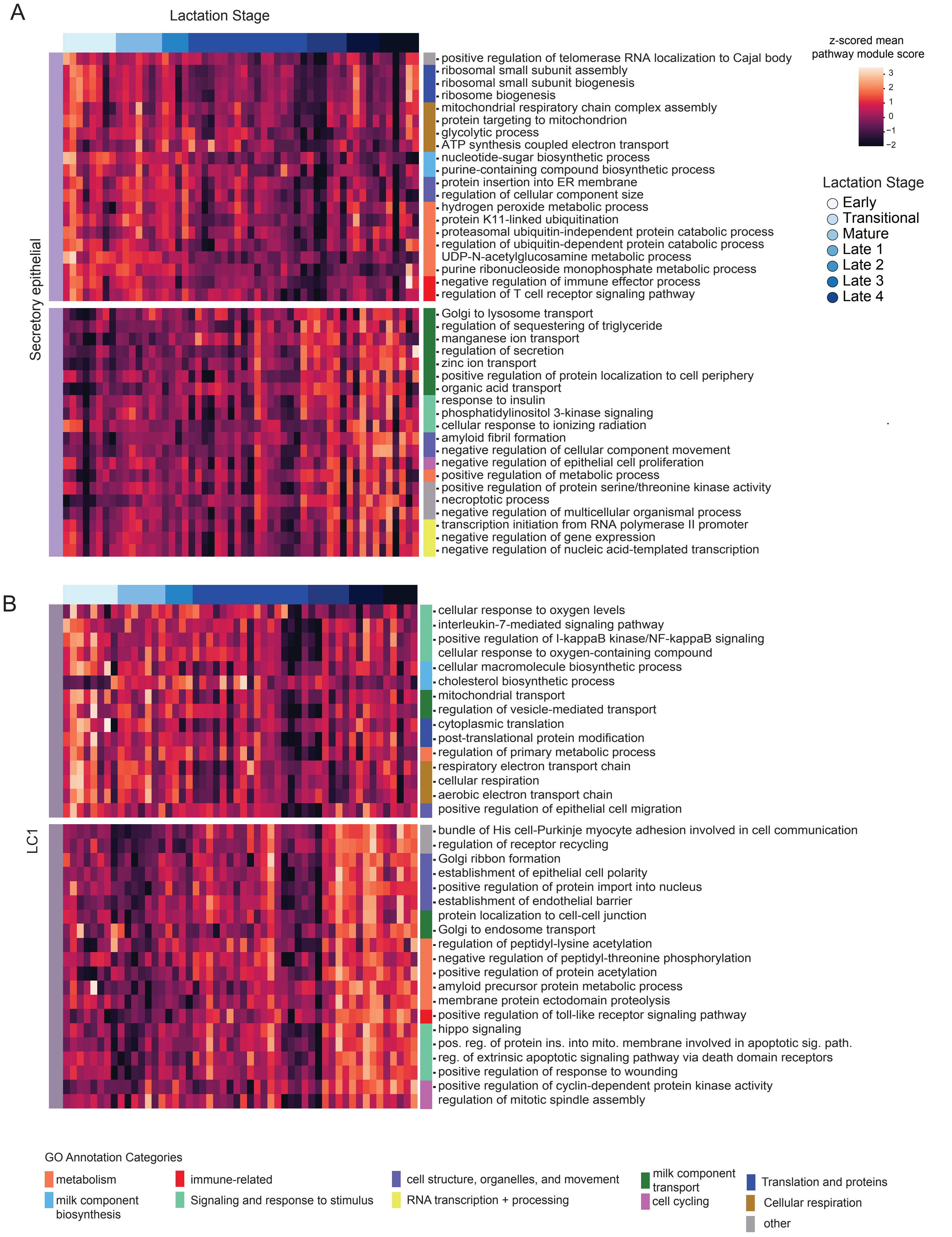
A
Lactation Stage
z-scored mean pathway module score
3
2
1
0
−1
−2
Lactation Stage
Early
Transitional
Mature
Late 1
Late 2
Late 3
Late 4
Secretory epithelial
positive regulation of telomerase RNA localization to Cajal body
ribosomal small subunit assembly
ribosomal small subunit biogenesis
ribosome biogenesis
mitochondrial respiratory chain complex assembly
protein targeting to mitochondrion
glycolytic process
ATP synthesis coupled electron transport
nucleotide-sugar biosynthetic process
purine-containing compound biosynthetic process
protein insertion into ER membrane
regulation of cellular component size
hydrogen peroxide metabolic process
protein K11-linked ubiquitination
proteasomal ubiquitin-independent protein catabolic process
regulation of ubiquitin-dependent protein catabolic process
UDP-N-acetylglucosamine metabolic process
purine ribonucleoside monophosphate metabolic process
negative regulation of immune effector process
regulation of T cell receptor signaling pathway
Golgi to lysosome transport
regulation of sequestering of triglyceride
manganese ion transport
regulation of secretion
zinc ion transport
positive regulation of protein localization to cell periphery
organic acid transport
response to insulin
phosphatidylinositol 3-kinase signaling
cellular response to ionizing radiation
amyloid fibril formation
negative regulation of cellular component movement
negative regulation of epithelial cell proliferation
positive regulation of metabolic process
positive regulation of protein serine/threonine kinase activity
necroptotic process
negative regulation of multicellular organismal process
transcription initiation from RNA polymerase II promoter
negative regulation of gene expression
negative regulation of nucleic acid-templated transcription
B
LC1
cellular response to oxygen levels
interleukin-7-mediated signaling pathway
positive regulation of I-kappaB kinase/NF-kappaB signaling
cellular response to oxygen-containing compound
cellular macromolecule biosynthetic process
cholesterol biosynthetic process
mitochondrial transport
regulation of vesicle-mediated transport
cytoplasmic translation
post-translational protein modification
regulation of primary metabolic process
respiratory electron transport chain
cellular respiration
aerobic electron transport chain
positive regulation of epithelial cell migration
bundle of His cell-Purkinje myocyte adhesion involved in cell communication
regulation of receptor recycling
Golgi ribbon formation
establishment of epithelial cell polarity
positive regulation of protein import into nucleus
establishment of endothelial barrier
protein localization to cell-cell junction
Golgi to endosome transport
regulation of peptidyl-lysine acetylation
negative regulation of peptidyl-threonine phosphorylation
positive regulation of protein acetylation
amyloid precursor protein metabolic process
membrane protein ectodomain proteolysis
positive regulation of toll-like receptor signaling pathway
hippo signaling
pos. reg. of protein ins. into mito. membrane involved in apoptotic sig. path.
reg. of extrinsic apoptotic signaling pathway via death domain receptors
positive regulation of response to wounding
positive regulation of cyclin-dependent protein kinase activity
regulation of mitotic spindle assembly
GO Annotation Categories
metabolism
milk component biosynthesis
immune-related
Signaling and response to stimulus
cell structure, organelles, and movement
RNA transcription + processing
milk component transport
cell cycling
Translation and proteins
Cellular respiration
other

Fig. S10. A. Reduced top Enrichr GO biological process results on genes changing over time in secretory epithelial cluster and **B**. LC1 epithelial cluster heatmaps represent sample means of gene set scores of each pathway (rows) z-scored across samples (columns), samples ordered by increasing time postpartum. Pathways colored by curated related GO term classifications, full results in Dataset S9.

Table S1: Donor information

Donor	Age	Week Delivered	Labor Induced	Antibiotics During Delivery	Pregnancy Number	Delivery Mode	Race	height (from forms)	height (inches)	height (m)	estimated weight over the course of the study (lbs)	estimated pre-pregnancy weight (lbs)	estimated weight over the course of the study (kg)	estimated pre-pregnancy weight (kg)	BMI (over course of study)	BMI (pre-pregnancy)
BM01	31	42	N	N	1	vaginal	caucasian	5'2"	62	1.57	130	121	59.04	54.96	23.81	22.16
BM02	31	42	Y	N	1	vaginal	caucasian	5'7"	67	1.70	130	126	58.82	57.30	20.31	19.79
BM03	31	37	N	Y	1	vaginal	caucasian	172 cm	68	1.72	154	134	70.00	61.00	23.66	20.62
BM04	35	42	N	Y	1	vaginal	caucasian	5'3"	63	1.60	188	192	85.28	87.09	33.30	34.01
BM05	31	39	Y	Y	1	vaginal	asian	5'9"	69	1.75	170	135	77.25	61.23	25.15	19.94
BM06	32	41	N	Y	1	vaginal	caucasian	5'3"	63	1.60	107	106	48.38	48.23	18.89	18.84
BM07	34	39	N	Y	3	C section	european	5'5"	65	1.65	170	155	77.26	70.31	28.34	25.79
BM08	31	38	N	N	1	C section	caucasian	5'7"	67	1.70	147	142	66.68	64.41	23.02	22.24
BM09	33	40	Y	Y	3	C section	caucasian	5'1"	61	1.55	195	187	88.45	84.82	36.84	35.33
BM10	31	41	Y	N	1	C section	caucasian	5'0"	60	1.52	152	125	69.10	56.70	29.75	24.41
BM11	33	39	N	N	1	vaginal	caucasian	5'8"	68	1.73	145	145	65.62	65.77	22.00	22.05
BM12	34	39	Y	N	1	vaginal	caucasian	5'7"	67	1.70	170	178	76.96	80.74	26.57	27.88
BM13	33	42	Y	Y	1	C section	hispanic/white	5'6"	66	1.68	154	152	69.85	68.95	24.86	24.53
BM15	34	37	Y	Y	1	vaginal	caucasian	5'3"	63	1.60	140	160	63.50	72.57	24.80	28.34
BM16	30	40	Y	Y	1	vaginal	caucasian	5'5"	65	1.65	135	135	61.23	61.23	22.46	22.46
BM17	25	42	Y	N	1	vaginal	caucasian	na	na	na	na	na	na	na	na	na
BM19	29	42	N	N	2	vaginal	caucasian	5'6"	66	1.68	185	155	83.91	70.31	29.86	25.02

Table S2: Generalized additive model (GAM) results for celltype proportion association with sample metadata

**p-values of association of each celltype proportion with metadata using generalized additive model with formula proportion ~ donor + <metadata_column>**						
	time post partum	any_formula_Yes	daycare_Yes	hormonal_birthcontrol_yes	hormonal_birthcontrol_no	vaccines_no
B Cells	-	-	-	-	-	-
T Cells	0.00234	-	-	-	-	-
Dendritic Cells	-	-	-	-	0.01705	0.03
Eosinophils	-	-	-	-	-	-
LC1	0.000189	-	0.03521	0.00532	-	-
LC2	-	-	-	-	-	-
GPNMB+ Macrophage	-	0.026	-	-	-	-
CSN1S1+ Macrophage	-	-	-	-	-	-
Neutrophils	-	-	-	-	-	-
**Number of samples included in comparison**	56	55	55	53	53	47

**p-values of association of each celltype proportion with each donor using generalized additive model with formula proportion ~ donor+s(time_post_partum)**													
	BM01	BM02	BM03	BM04	BM05	BM06	BM07	BM08	BM10	BM11	BM12	BM16	BM19
B Cells	-	-	-	-	-	-	-	2.53E-07	-	-	-	-	-
T Cells	0.01911	-	-	0.01045	-	0.00326	-	0.00659	0.00339	-	-	-	-
Dendritic Cells	-	-	-	-	0.0422	-	-	0.00276	-	-	-	-	-
Eosinophils	-	-	-	-	-	-	-	-	-	-	-	-	-

Fibroblasts	-	-	-	-	-	-	-	-	-	-	-	0.00265	-
LC1	0.000918	-	-	-	-	0.049171	-	-	-	-	-	0.002811	-
LC2	1.72E-11	-	-	-	0.000191	-	-	0.022375	-	-	-	-	-
GPNMB+ Macrophage	-	-	-	-	7.55E-06	-	-	-	-	-	-	0.0138	-
CSN1S1+ Macrophage	-	-	-	-	1.17E-09	-	0.0118	-	-	-	-	-	-
Neutrophils	-	-	-	-	-	-	-	-	-	-	-	-	-

p-values of association of each immune celltype proportion with metadata using generalized additive model with formula proportion ~ donor + <metadata_column>

	time postpartum	any_formula_no	Infant_sick_yes	vaccines_no
B Cells	-	-	-	-
T Cells	0.0541	-	-	-
Dendritic Cells	-	-	0.0117	-
Eosinophils	-	0.0120	-	-
GPNMB+ Macrophage	-	-	-	0.0302
CSN1S1+ Macrophage	-	-	-	-
Neutrophils	-	-	-	-
**Number of samples included in comparison**	56	55	53	47

p-values of association of each celltype proportion with each donor using generalized additive model with formula proportion ~ donor+s(time_post_partum)

	BM01	BM02	BM03	BM04	BM05	BM06	BM07	BM08	BM10	BM11	BM12	BM16	BM19
B Cells	-	-	-	-	-	-	-	0.0178	-	-	-	-	-
T Cells	-	-	-	-	-	-	-	-	-	-	-	-	-
Dendritic Cells	-	-	-	-	-	-	-	-	-	-	-	0.0412	-
Eosinophils	-	-	-	-	-	-	-	-	-	-	-	-	-
GPNMB+ Macrophage	-	-	-	-	0.000149	-	-	-	-	-	-	-	-
CSN1S1+ Macrophage	-	-	-	-	0.00035	-	-	-	-	-	-	-	-
Neutrophils	-	-	-	-	-	-	-	-	-	-	-	-	-

p-values of association of each epithelial celltype proportion with metadata using generalized additive model with formula proportion ~ donor + <metadata_column>

	time post partum	daycare_Yes	hormonal_birthcontrol_yes
Cycling lactocytes	-	-	-
KRT high 1	-	-	-
KRT high 2	0.049	-	-
LC1	0.00335	-	-
MT High LC2	-	0.0139	0.00835
Secretory lactocytes	-	-	-
**Number of samples included in comparison**	55	54	52

p-values of association of each celltype proportion with each donor using generalized additive model with formula proportion ~ donor+s(time_post_partum)

	BM01	BM02	BM03	BM04	BM05	BM06	BM07	BM08	BM10	BM11	BM12	BM16	BM19
Cycling lactocytes	-	-	-	-	-	-	0.03235	-	-	-	0.00075	-	0.00293
KRT high 1	-	-	-	-	-	-	-	-	1.72e-5	-	-	-	-
KRT high 2	-	-	-	-	-	-	-	-	-	-	-	-	-
LC1	0.00131	-	-	-	-	-	-	-	-	-	-	0.00174	-
MT High LC2	-	-	-	-	-	-	-	-	-	-	-	-	-
Secretory lactocytes	-	-	-	-	-	-	-	-	-	-	-	-	-

Dataset S1 (separate file): Sample-level metadata
Dataset S2 (separate file): All cells marker genes
Dataset S3 (separate file): Macrophage sub-cluster marker genes
Dataset S4 (separate file): Macrophage Reactome results
Dataset S5 (separate file): Lactocyte and monocyte gene modules
Dataset S6 (separate file): Epithelial cell marker genes
Dataset S7 (separate file): Time-associated genes for each epithelial cell subcluster
Dataset S8 (separate file): Universal epithelial varying genes and genes which change in opposite directions with time in LC1 epithelial cells and secretory epithelial cells
Dataset S9 (separate file): Enrichr results for time-associated genes in epithelial cells

SI References

1. Z. Fang, *GSEApy: Gene Set Enrichment Analysis in Python* (Zenodo, 2020) https:/doi.org/10.5281/ZENODO.3748085 (September 16, 2021).
2. M. Ashburner, *et al.*, Gene Ontology: tool for the unification of biology. *Nat. Genet.* **25**, 25–29 (2000).
3. F. A. Wolf, P. Angerer, F. J. Theis, SCANPY: large-scale single-cell gene expression data analysis. *Genome Biol.* **19**, 15 (2018).
4. A.-J. Twigger, *et al.*, Transcriptional changes in the mammary gland during lactation revealed by single cell sequencing of cells from human milk. *Nat. Commun.* **562** (2022).
5. J. F. Martin Carli, *et al.*, Single Cell RNA Sequencing of Human Milk-Derived Cells Reveals Sub-Populations of Mammary Epithelial Cells with Molecular Signatures of Progenitor and Mature States: a Novel, Non-invasive Framework for Investigating Human Lactation Physiology. *J. Mammary Gland Biol. Neoplasia* **25**, 367–387 (2020).
6. L. M. Murrow, *et al.*, "Changes in epithelial proportions and transcriptional state underlie major premenopausal breast cancer risks" (bioRxiv, 2020) https:/doi.org/10.1101/430611 (August 23, 2021).
